# *ESX1* gene as a potential candidate responsible for male infertility in nonobstructive azoospermia

**DOI:** 10.1038/s41598-023-43854-9

**Published:** 2023-10-02

**Authors:** Agnieszka Malcher, Zuzanna Graczyk, Hermann Bauer, Tomasz Stokowy, Andrea Berman, Mikołaj Smolibowski, Dominika Blaszczyk, Piotr Jedrzejczak, Alexander N. Yatsenko, Maciej Kurpisz

**Affiliations:** 1 Institute of Human Genetics, Polish Academy of Sciences, Poznan, Poland; 2 Department of Developmental Genetics, Max Planck Institute for Molecular Genetics, Berlin, Germany; 3 IT Division, University of Bergen, Bergen, Norway; 4 Department of Biological Sciences, University of Pittsburgh, Pittsburgh, USA; 5 Department of Cell Biology, Center of Obstetrics, Gynecology and Infertility Treatment, University of Medical Sciences, Poznan, Poland; 6 Department of OB/GYN and Reproductive Sciences, School of Medicine, University of Pittsburgh, Pittsburgh, PA USA

**Keywords:** Preclinical research, Biological techniques, Bioinformatics, Biological models

## Abstract

Infertility is a problem that affects approximately 15% of couples, and male infertility is responsible for 40–50% of these cases. The cause of male infertility is still poorly diagnosed and treated. One of the prominent causes of male infertility is disturbed spermatogenesis, which can lead to nonobstructive azoospermia (NOA). Whole-genome sequencing (WGS) allows us to identify novel rare variants in potentially NOA-associated genes, among others, in the *ESX1* gene. The aim of this study was to activate the *ESX1* gene using CRISPRa technology in human germ cells (testicular seminoma cells—TCam-2). Successful activation of the *ESX1* gene in TCam-2 cells using the CRISPRa system was achieved, and the expression level of the *ESX1* gene was significantly higher in modified TCam-2 cells than in WT cells or the negative control with nontargeted gRNA (*p* < 0.01). Using RNA-seq, a network of over 50 genes potentially regulated by the *ESX1* gene was determined. Finally, 6 genes, *NANOG, CXCR4, RPS6KA5, CCND1, PDE1C,* and *LINC00662*, participating in cell proliferation and differentiation were verified in azoospermic patients with and without a mutation in the *ESX1* gene as well as in men with normal spermatogenesis, where inverse correlations in the expression levels of the observed genes were noted.

## Introduction

Infertility is a problem that affects approximately 15% of couples. In 45% of such couples, male infertility can be involved^1^. The causes of male infertility are still poorly diagnosed. One of the reasons for male infertility is disturbed spermatogenesis, which can be manifested as nonobstructive azoospermia. In more than 40% of men with impaired spermatogenesis, the aetiology remains unknown after the standard diagnostic tests are performed, although the molecular background can be envisaged^2,3^. The use of high-throughput genome-wide systemic analysis tools, such as next-generation sequencing (NGS) and international consortium projects (e.g., HapMap, 1000 Genomes Project), allowed genomic research at a large scale. The results obtained with these methods provided a better understanding of complex civilization diseases, e.g., cancer or cardiomyopathy^4^ and allowed the identification of a large number of mutations in novel genes potentially responsible for the NOA phenotype, such as *TEX11* (testis-expressed 11)^5,6^, *M1AP* (meiosis 1-associated protein)^7^, *TDRD9* (Tudor domain-containing 9)^8^, *GCNA* (germ-cell nuclear antigen)^9^, *GTF2H3* (general transcription factor TFIIH subunit 3)^10^, *MEIOB* (meiosis specific with OB-fold)^5^, and *MEI1* (meiotic double-stranded break formation protein 1)^5^. However, most of NOA cases remain undiagnosed, and their genomic aetiology still need more studies to be characterized.

In our previous research using whole-genome sequencing (WGS), we detected two ultrarare variants, *chrX:103495090G* > *C/c.1040C* > *G/p.Pro347Arg* and *chrX:103495088G* > *C*/*c.1042C* > *G/p.Leu348Val* (according to GRCh37), in the *ESX1* gene (extraembryonic spermatogenesis homeobox 1) in 2 out of 39 patients with nonobstructive azoospermia^11^. These are complex mutations because both variants occur in the X chromosome, which is present in only one copy per cell in males. In addition to our findings^11^, the association between ESX1 variations and NOA was also shown by the Chinese group^12^, who selected novel variants of *ESX1* within 766 NOA patients and 709 fertile controls^12^. They have identified similar variants to ours in proline rich repeat region, but more closer to the end of this region (p.Pro365Arg and p.Leu366Val) and found that the compound variant compromised the stabilizing effect of ESX1 on cyclin A, thereby causing the failure of M phase arrest in cells^12^.

The human *ESX1* gene is present on the X chromosome at position Xq22.2 and has 4 exons. It is a homeotic gene that is only expressed in the male gonad of adult males^13,14^. This gene, which encodes a dual-function 65 kDa protein, contains a homeodomain in the N-terminal fragment and a proline-rich region in the C-terminal fragment^15^. It has been reported that the *ESX1* transcript in the semen of men with NOA is a suitable molecular marker for predicting the presence of residual foci of spermatogenesis in the testis^16^. Moreover, ESX1 may inhibit cyclin degradation^15^ and may be involved in the regulation of cell cycle progression during human spermatogenesis^12^.

Here, we present studies of the *ESX1* gene using the CRISPR (Clustered Regularly Interspaced Short Palindromic Repeats) activation system in the testicular seminoma cell line TCam-2 as the first step to investigate its potential function regarding gene cluster regulated by *ESX1*. In this study we also shown that the previously identified variants lead to amino acid sequence changes in the proline-rich repeat region (PRR) of the ESX1 protein which may affect the correct folding of the protein. As PRRs have been observed to bind the major groove of DNA, the identified mutations: c.1040C > G (p.Pro347Arg) and c.1042C > G (p.Leu348Val) are predicted to affect the overall regular helical structure inherent in the PRR of ESX1, thereby potentially affecting sequence-specific interactions.

## Results

### Sanger validation of identified SNVs (single nucleotide variants) from WGS

In our previous published studies using whole genome sequencing—WGS analysis, we identified two ultrarare variants in the *ESX1* gene (c.1040C > G, c.1042C > G) presented as compound mutation in 2 patients with NOA at the postmeiotic arrest phase, as detailed by Malcher et al.^11^. Using Sanger sequencing, we confirmed the obtained SNVs (single nucleotide variants) from WGS analysis of these patients (Supplementary Fig. 1).

### Structural protein analysis of ESX1

**Figure 1:**
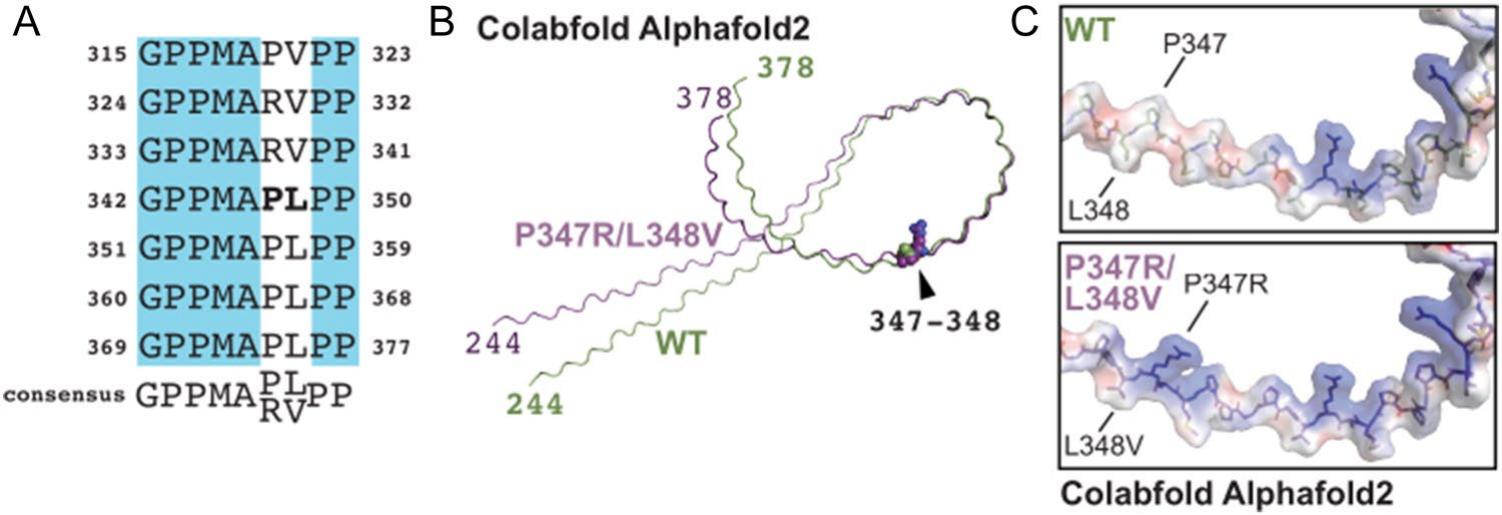
The proline-rich region of ESX1 likely forms polyproline (II) helices. (**A**) Alignment of the last seven repeats of the proline-rich region of ESX1. (**B**) Alignment of the ColabFold AlphaFold2-generated structures of the proline-rich regions of the wild-type and P347R/L348V ESX1 variants shown in ribbon representation. The N- and C-termini are labelled. Residues at positions 347 and 348 are shown as spheres. (**C**) Zoomed view of the surface representation of the region surrounding residues 347 and 348 modelled by ColabFold AlphaFold2. The surface is coloured by electrostatic potential as calculated in PyMOL; blue, positive; red, negative (~ − 30–30 eV). The compound mutation of p.Pro347Arg/p.Leu348Val would be expected to affect its ability to interact with binding partners.

The three-dimensional structural model of the homeobox protein ESX1 generated by the machine-learning-based AlphaFold (Deepmind Technologies, Ltd.)^17^ indicates several regions of disorder surrounding the confidently modelled homeobox domain (data not shown). The residues of interest fall within a C-terminal proline-rich region consisting of a nine-residue P-P-x-x-P-x-P-P-x motif repeated fifteen times^15,18^, spanning amino acids 244–378 (Fig. 1A). We used ColabFold AlphaFold2 and ColabFold AlphaFold2_advanced_beta to model this region^17,19^. As expected, both programs generated polyproline II-type structures (Fig. 1B,C and Supplementary Fig. 2). This secondary structure has three amino acids per left-hand turn, creating a fairly rigid structural element^20,21^. Proline-rich regions foster protein‒protein interactions, including the recruitment, stabilization, and scaffolding of protein complexes^20,21^. Consistent with these findings, ColabFold AlphaFold2 predicted regions of disorder surrounding the homeobox domain of ESX1^17^, although disordered regions are difficult to predict accurately.

The p.Pro347Arg/p.Leu348Val compound mutation could affect several aspects of the larger structure. First, it inverts the surface potential of the region from negative to positive (Fig. 1C). The atomic shape of this area of the polyproline helix would also change; the bulky side chain of proline is topologically linked to the polypeptide backbone, limiting the rotational space that the residue can occupy. Thus, these mutations would be expected to affect its ability to interact with binding partners.

### Localization of the *ESX1* gene in human testis

**Figure 2:**
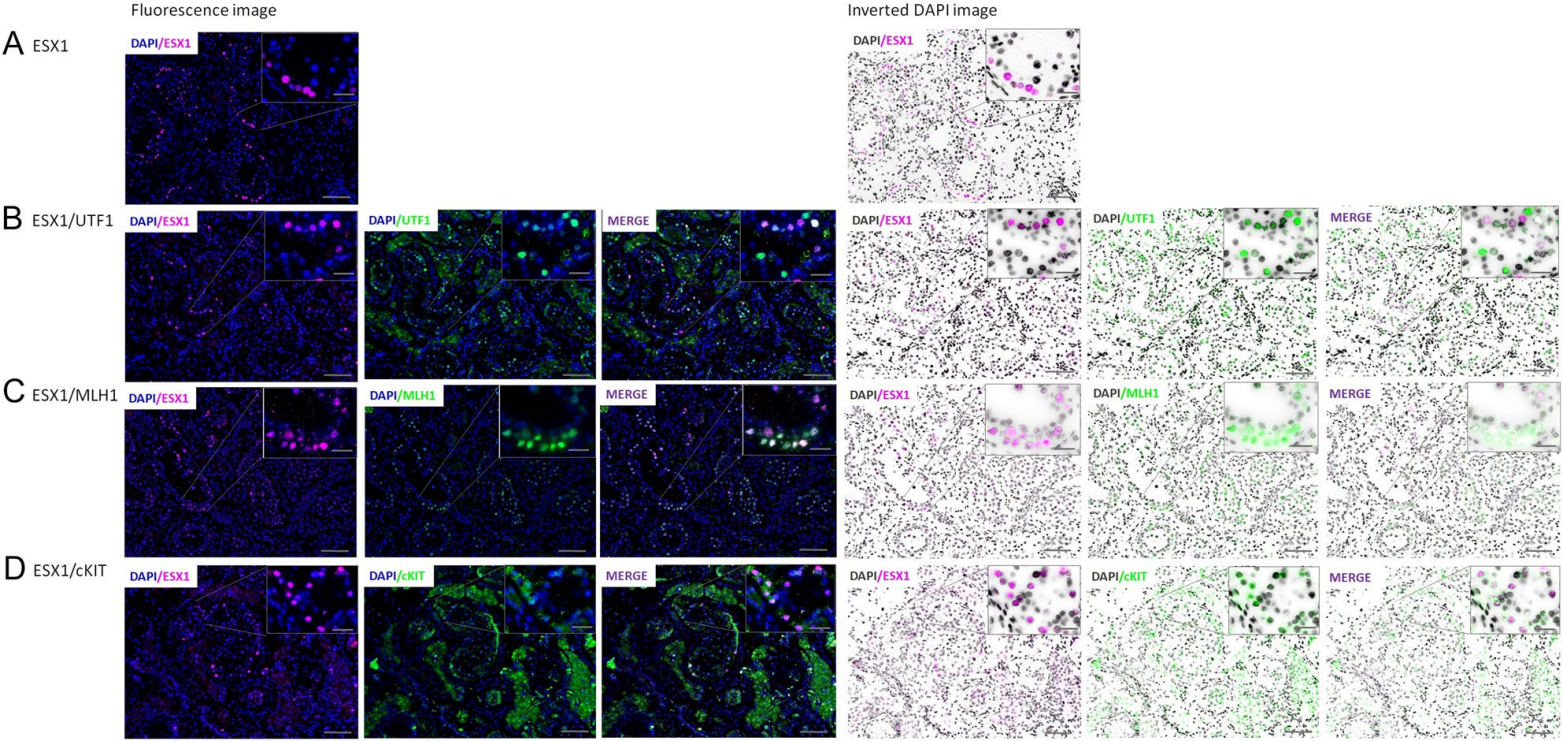
Immunofluorescence and inverted DAPI image of ESX1 localization in normal human testis. (**A**) Staining of ESX1. (**B**) Co-staining of ESX1 with UTF1. (**C**) Co-staining of ESX1 with MLH1. (**D**) Co-staining of ESX1 with c-KIT. Leica DM5500 with a proper filter set (DAPI/SpO/Triple) was used; objectives: 10x, scale bar: 100 µm, and 63x, scale bar: 20 µm with oil immersion; software: CytoVision. Immunofluorescence analysis showed that ESX1 protein is produced mostly in the nucleus of state 1, 2, 3 and 4 spermatogonia in normal adult human testis.

Immunofluorescence analysis of control testicular tissue showed that ESX1 protein is produced mostly in the nucleus of spermatogonia in normal adult human testis (Fig. 2A–D). In the co-staining of ESX1 with UTF1, most of the nuclear UTF1-positive undifferentiated spermatogonia presented strongly stained ESX1 protein, however, there were also cells strongly positive only for ESX1 or exclusively for UTF1 (Fig. 2B). In the case of ESX1 co-stained with MLH1, we observed mostly the same positive-stained nuclear spermatogonia for both proteins and only some were strongly positive exclusively for ESX1 or for MLH1 protein (Fig. 2C). Double staining of the ESX1 and c-KIT proteins presented cytoplasmatic localization for c-KIT and nucleus for ESX1 in differentiated spermatogonia, but also there were some cells positive only for ESX1 protein (Fig. 2D). The c-KIT also showed a strong expression in Leydig cells.

### Activation of the *ESX1* gene by transfection of TCam-2 cells with the CRISPRa system

**Figure 3:**
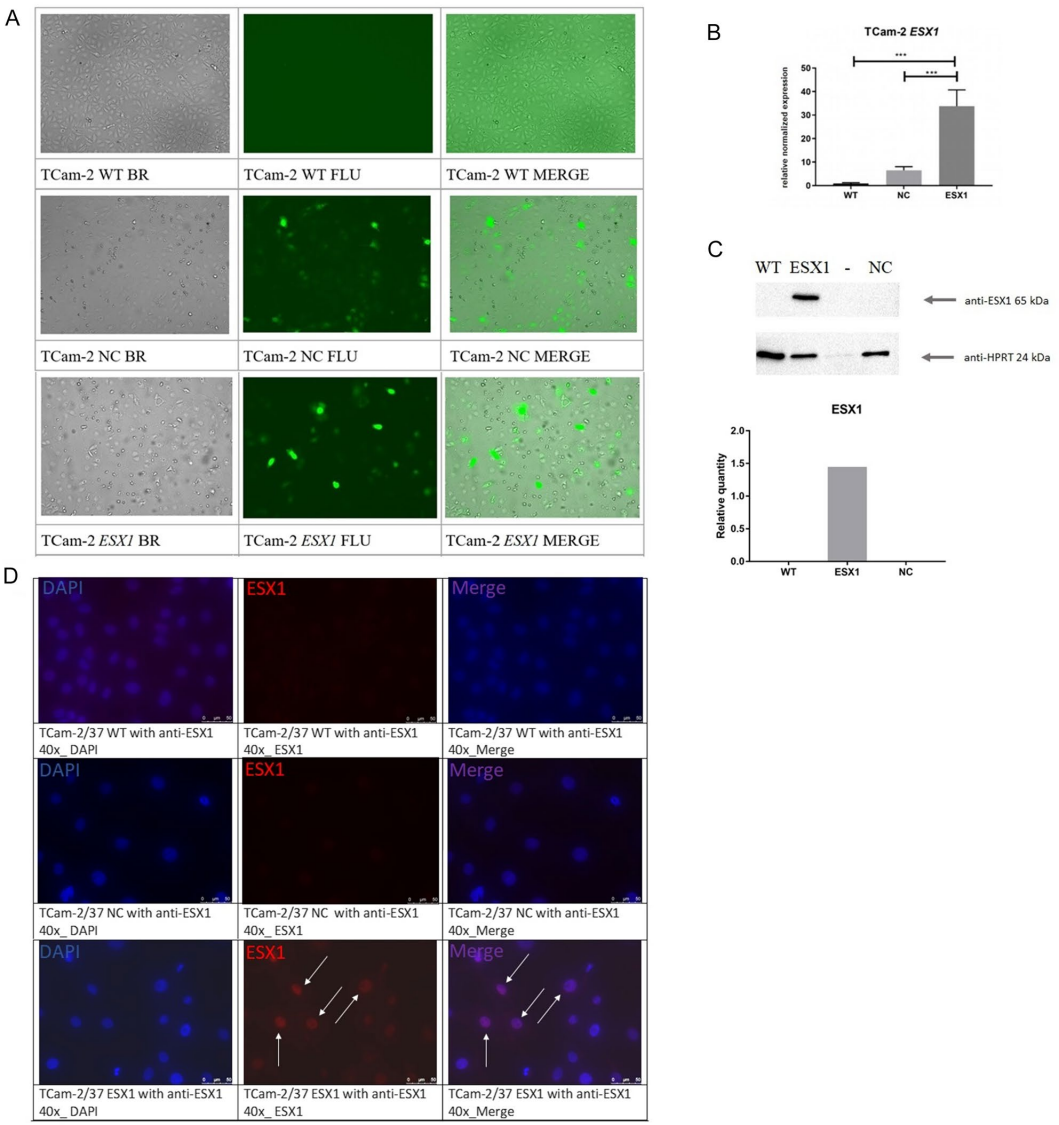
Activation of the *ESX1* gene using the CRISPRa system. (**A**) TCam-2 cells at 72 h after transfection under a Juli FL fluorescence microscope; scale bar: 100 μm. (**B**) Real-time PCR analysis of *ESX1* gene expression. (**C**) Representative Western blot analysis of ESX1 protein isolated from TCam-2 cells, exposure time 300 s; and its graph of the relative quantity of the ESX1 protein normalized with reference to HPRT1 analysed by Image Lab 6.1 tools. (**D**) Immunofluorescence analysis of TCam-2 cells. A Leica DMi8 with a proper filter set (DAPI/TxR/Triple) was used; objectives: 40x, scale bar: 50 μm; software: LASX. Arrows indicate examples of ESX1-positive staining. The transfection efficiency after antibiotic selection and induction with doxycycline was estimated for approximately 40% for both TCam-2—cells with the activated *ESX1* gene and the negative control—cells with nonspecific gRNAs (guide RNAs) for the human genome. Activation of the ESX1 gene in TCam-2 cells was successfully achieved at the mRNA level with significant upregulation (*p* < 0.001) of the *ESX1* gene in comparison to both applied control samples—WT and NC; and that was also observed at the protein level, where the presence of the ESX1 protein was observed in TCam-2 cells with active *ESX1*, while in WT and NC, the protein was absent. *WT* wild type, *NC* negative control with nonspecific gRNAs for the human genome, *ESX1* cells with the activated *ESX1* gene using specific gRNAs for the *ESX1* sequence, *BR* brightfield, *FLU* fluorescence.

The transfection efficiency was estimated after antibiotic selection and induction with doxycycline (after 72 h) and was approximately 40% of GFP-positive signal for both samples—TCam-2 with the activated *ESX1* gene—35% and the negative control—TCam-2 with nonspecific gRNAs (guide RNAs) for the human genome—42% (Fig. 3A). *ESX1* gene activation was determined after 72 h at the mRNA level using real-time PCR (Fig. 3B) and at the protein level using Western blot and immunofluorescence staining (Fig. 3C,D). We observed significant upregulation (*p* < 0.001) of the *ESX1* gene in TCam-2 samples with the activated *ESX1* gene (marked as ESX1) in comparison to both control groups—TCam-2 wild type (WT) and the negative control, which was TCam-2 with nonspecific gRNAs for the human genome (NC) (Fig. 3B). We also confirmed ESX activation at the protein level in Western blot analysis, where we observed the presence of the ESX1 protein, while in wild-type and negative controls, the protein was absent (Fig. 3C). Additionally, by using immunofluorescent staining, we observed the presence of ESX1 protein with very strong (47%) and weak signals (53%) in the nuclei of modified cells with gRNA for the *ESX1* gene, while in both applied controls, no protein signal was detected (Fig. 3D).

### RNA sequencing (RNA-seq) analysis

**Figure 4:**
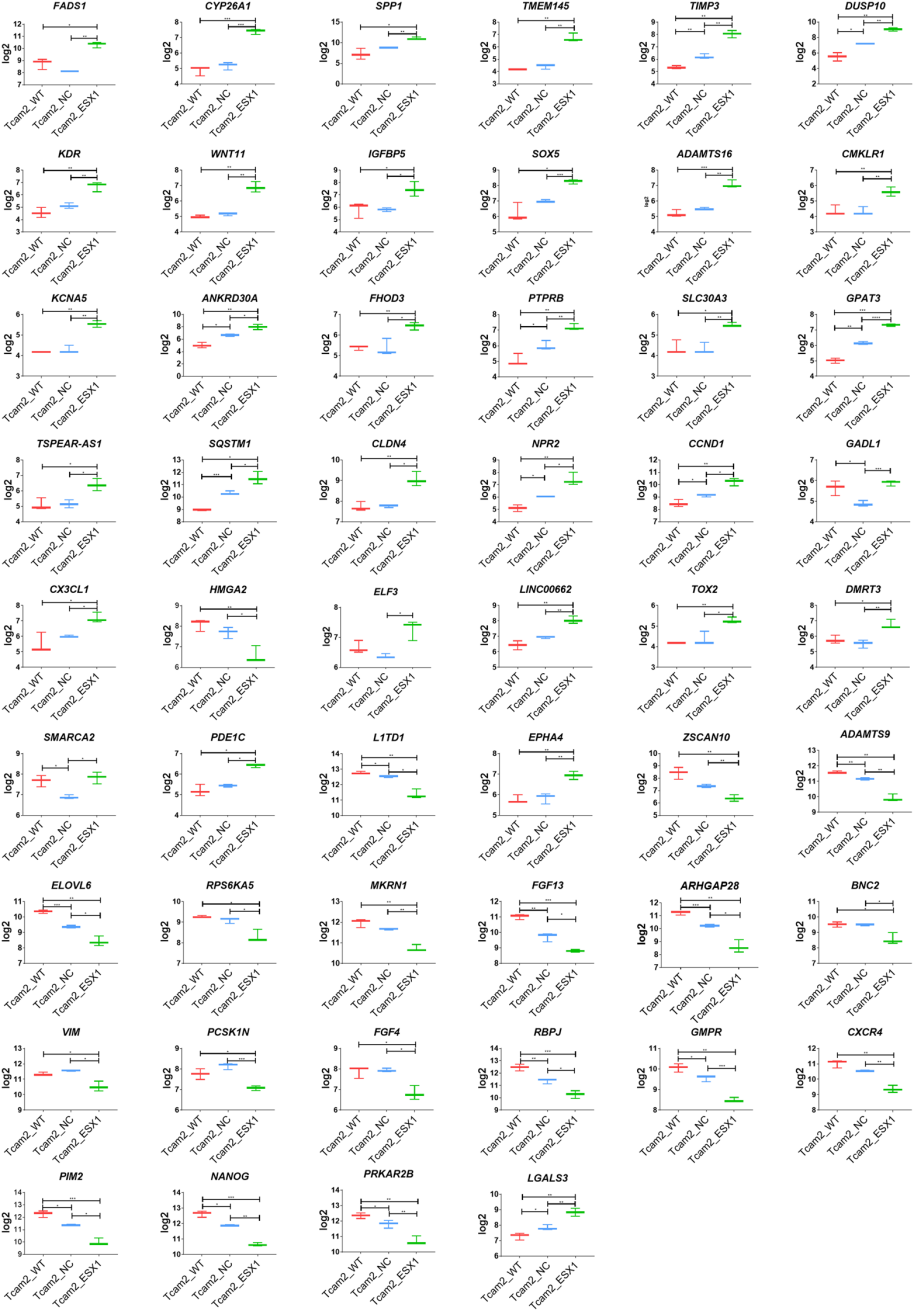
Box-plot graphs of 52 selected genes analysed in TCam-2 cells with activated *ESX1* gene and controls analysed by RNA-seq. The 33 out of the selected genes presented upregulation and 19 downregulation with a minimum of a twofold change (*p* < 0.05) in cells with activated ESX1 in comparison to both applied controls. *WT* wild type, *NC* negative control with nonspecific gRNAs for the human genome, *ESX1* cells with the activated *ESX1* gene using specific gRNAs for the *ESX1* sequence. *p < 0.05, **p < 0.01, ***p < 0.001, ****p < 0.0001.

First, we have confirmed the activation of the *ESX1* gene in TCam-2 cells by RNA sequencing (Supplementary Fig. 3A). Then, based on a minimum twofold change and a *p* value below 0.05 to determine the relationship with spermatogenesis, we selected over 50 genes that differed significantly in expression (*p* < 0.05) between the TCam-2 cells modified by the CRISPRa system with specific gRNAs for the *ESX1* gene in comparison to both controls (Supplementary Fig. 3B,C). The genes *FADS1* (fatty acid desaturase 1)*, CYP26A1* (cytochrome P450 family 26 subfamily A member 1)*, SPP1* (secreted phosphoprotein 1)*, TMEM145* (transmembrane protein 145)*, TIMP3* (TIMP metallopeptidase inhibitor 3)*, DUSP10* (dual specificity phosphatase 10)*, KDR* (kinase insert domain receptor)*, WNT11* (Wnt family member 11)*, IGFBP5* (insulin like growth factor binding protein 5)*, SOX5* (SRY-box transcription factor 5)*, ADAMTS16* (ADAM metallopeptidase with thrombospondin type 1 motif 16)*, CMKLR1* (chemerin chemokine-like receptor 1)*, KCNA5* (potassium voltage-gated channel subfamily A member 5)*, ANKRD30A* (ankyrin repeat domain 30A)*, FHOD3* (formin homology 2 domain containing 3)*, PTPRB* (protein tyrosine phosphatase receptor type B)*, SLC30A3* (solute carrier family 30 member 3)*, GPAT3* (glycerol-3-phosphate acyltransferase 3)*, TSPEAR-AS1* (TSPEAR antisense RNA 1)*, SQSTM1* (sequestosome 1)*, CLDN4* (claudin 4)*, NPR2* (natriuretic peptide receptor 2)*, CCND1* (cyclin D1)*, GADL1* (glutamate decarboxylase like 1)*, CX3CL1* (C-X3-C motif chemokine ligand 1)*, LGALS3* (galectin 3)*, LINC00662* (long intergenic non-protein coding RNA 662)*, ELF3* (E74 like ETS transcription factor 3)*, TOX2* (TOX high mobility group box family member 2)*, DMRT3* (doublesex and mab-3 related transcription factor 3)*, SMARCA2* (SWI/SNF related, matrix associated, actin dependent regulator of chromatin, subfamily a, member 2)*, PDE1C* (phosphodiesterase 1C)*,* and *EPHA4* (EPH receptor A4) were upregulated with a minimum of a twofold change (*p* < 0.05) in cells with activated *ESX1*, and genes including *ZSCAN10* (zinc finger and SCAN domain containing 10)*, ELOVL6* (ELOVL fatty acid elongase 6)*, RPS6KA5* (ribosomal protein S6 kinase A5)*, MKRN1* (makorin ring finger protein 1)*, FGF13* (fibroblast growth factor 13)*, ARHGAP28* (Rho GTPase activating protein 28)*, BNC2* (basonuclin zinc finger protein 2)*, VIM* (vimentin)*, PCSK1N* (proprotein convertase subtilisin/kexin type 1 inhibitor)*, FGF4* (fibroblast growth factor 4)*, RBPJ* (recombination signal binding protein for immunoglobulin kappa J region)*, GMPR* (guanosine monophosphate reductase)*, CXCR4* (C-X-C motif chemokine receptor 4)*, PIM2* (Pim-2 proto-oncogene, serine/threonine kinase)*, NANOG* (Nanog homeobox)*, PRKAR2B* (protein kinase cAMP-dependent type II regulatory subunit beta)*, L1TD1* (LINE1 type transposase domain containing 1)*, HMGA2* (high mobility group AT-hook 2)*,* and *ADAMTS9* (ADAM metallopeptidase with thrombospondin type 1 motif 9) were downregulated (*p* < 0.05) in the *ESX1-*activated cells in comparison to controls (Fig. 4). STRING analysis showed that there were known interactions (mostly indirect) of *ESX1* with some of these selected genes, such as *ELF3, VIM, HMGA2, CCND1, LGALS3 NANOG, L1TD1, SOX5, KDR* and *CXCR4* (Supplementary Fig. 3D), which were mostly involved in signal transduction and proliferation processes as determined by Gene Ontology analysis (CPDB—ConsensusPathDB database) (Supplementary Table 1).

### Verification of the gene expression level in TCam-2 cells

**Figure 5:**
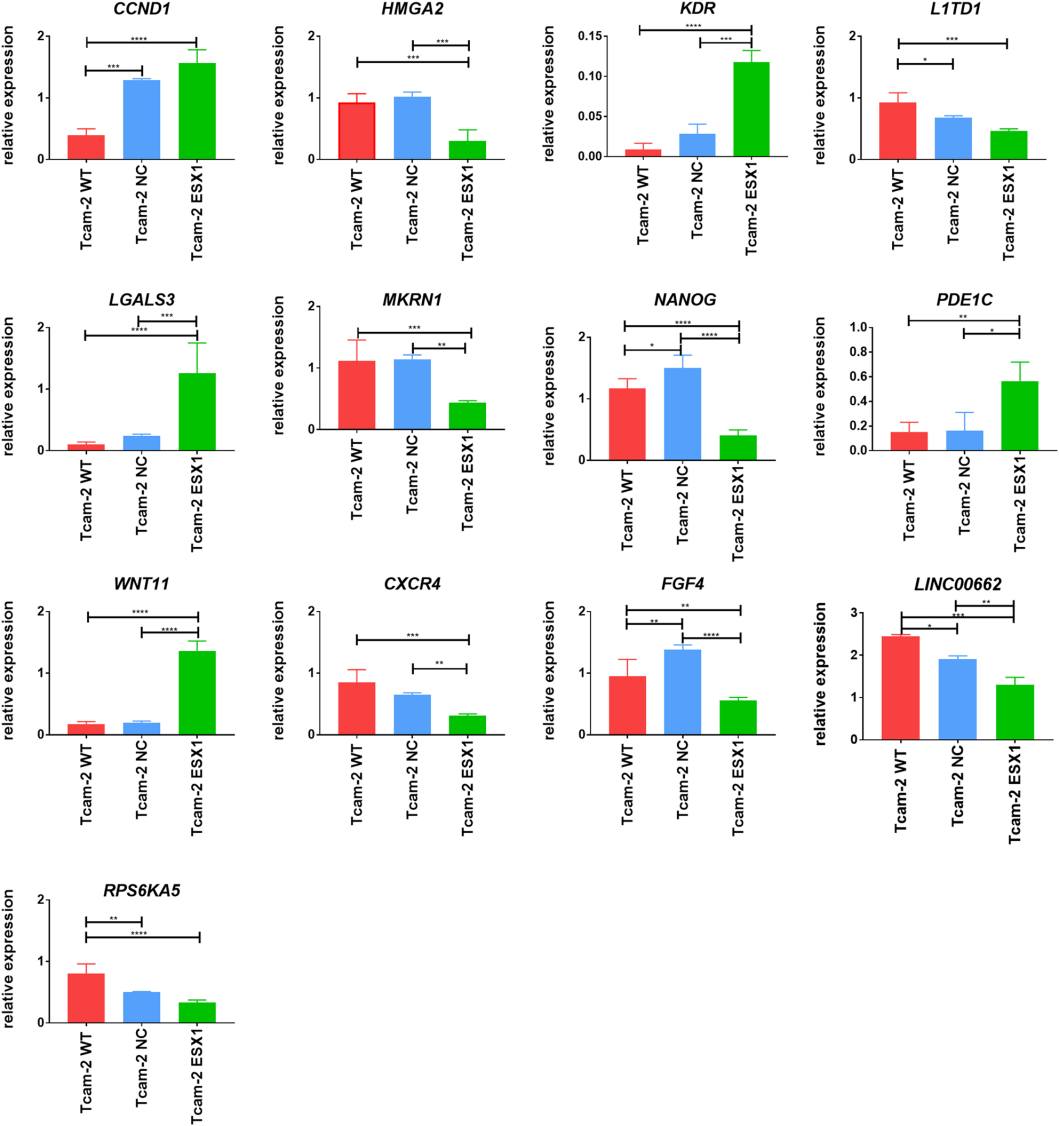
Verification of selected gene by real-time PCR *(CCND1, HMGA2, KDR, L1TD1, LGALS3, MKRN1, NANOG, PDE1C, WNT11, CXCR4, FGF4, LINC00662,* and *RPS6KA5)* in TCam-2 cells with the activated *ESX1* gene and controls. The *CCND1, KDR, LGALS3, PDE1C, WNT11* were upregulated (*p* < 0.05), and *HMGA2, L1TD1, MKRN1, NANOG, CXCR4, FGF4, RPS6KA5* were downregulated (*p* < 0.05) in TCam-2 cells with *ESX1* activation as compared to applied controls. *WT* wild type, *NC* negative control with nonspecific gRNAs for the human genome, *ESX1* cells with the activated *ESX1* gene using specific gRNAs for the *ESX1* sequence. *p < 0.05, **p < 0.01, ***p < 0.001, ****p < 0.0001.

Finally, we narrowed down the list of genes analysed by RNA-seq data and selected 13 key genes (*CCND1, HMGA2, KDR, L1TD1, LGALS3, MKRN1, NANOG, PDE1C, WNT11, CXCR4, FGF4, LINC00662,* and *RPS6KA5*) that were differentially expressed in cells with the activated *ESX1* gene compared to controls and verified them in the TCam-2 cell line by real-time PCR. *CCND1, KDR, LGALS3, PDE1C,* and *WNT11* were upregulated (*p* < 0.05), and *HMGA2, L1TD1, MKRN1, NANOG, CXCR4, FGF4, LINC00662* and *RPS6KA5* were downregulated (*p* < 0.05) in TCam-2 cells with *ESX1* activation compared to controls (Fig. 5).

### Verification of gene expression levels in NOA patients

**Figure 6:**
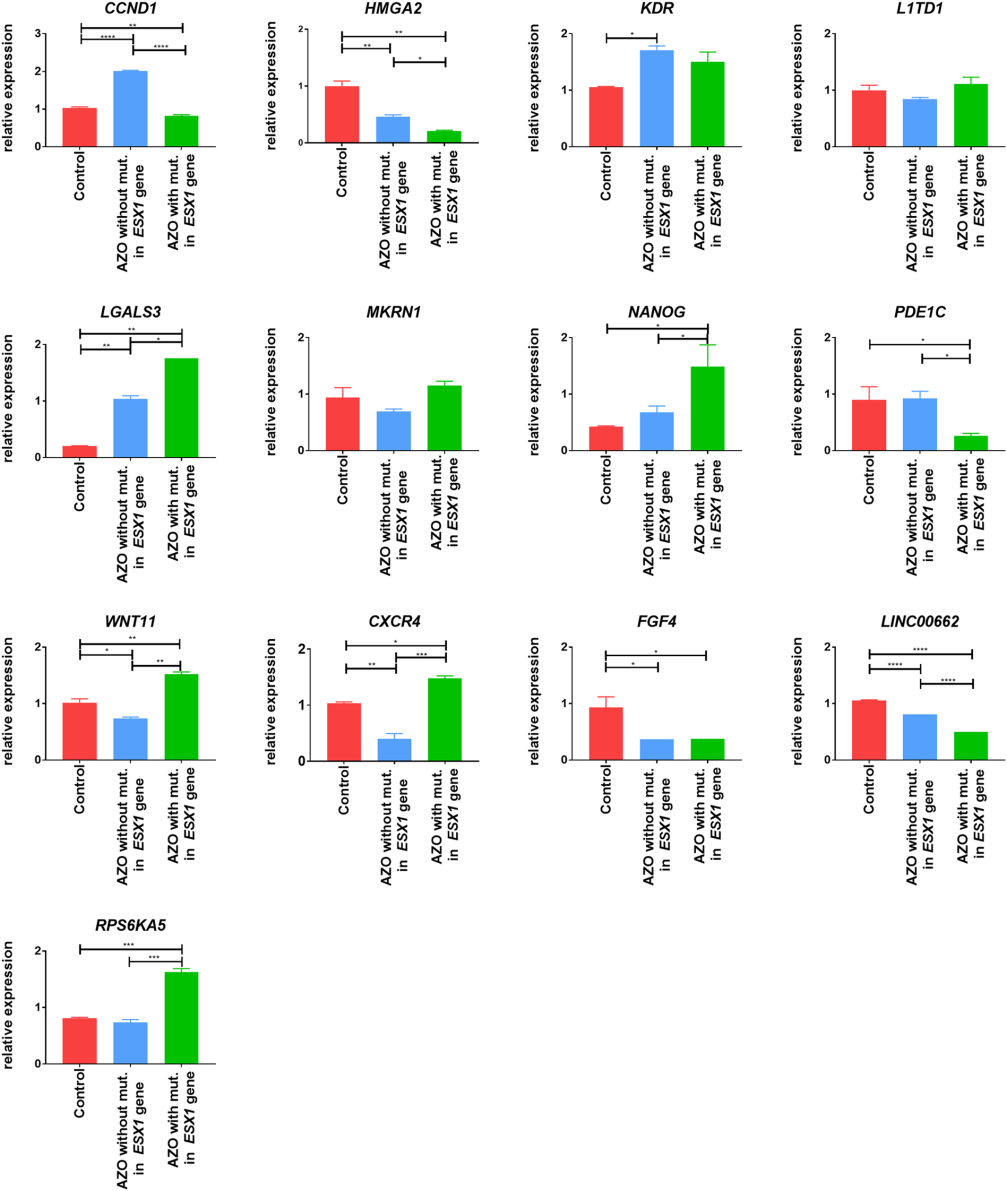
Verification of selected gene by real-time PCR (*CCND1, HMGA2, KDR, L1TD1, LGALS3, MKRN1, NANOG, PDE1C, WNT11, CXCR4, FGF4, LINC00662,* and *RPS6KA5*) in patients with NOA and controls. The *CCND1, HMGA2, PDE1C, LINC00662* genes were downregulated (*p* < 0.05), and *LGALS3, NANOG, WNT11, CXCR4, RPS6KA5* were upregulated (*p* < 0.05) in the male gonad tissue sample obtained from an azoospermic patient with a mutation in the ESX1 gene in comparison to an azoospermic patient without mutation in the *ESX1* gene and control. Control-male gonadal tissue from men with normal spermatogenesis; AZO without mut. in the ESX1 gene-patient with nonobstructive azoospermia at postmeiotic arrest without mutation in the *ESX1* gene; AZO with mut. in the *ESX1* gene- patient with nonobstructive azoospermia at postmeiotic arrest (10 L) with mutation in the *ESX1* gene. *p < 0.05, **p < 0.01, ***p < 0.001, ****p < 0.0001.

We also verified the 13 selected differentially expressed genes (*CCND1, HMGA2, KDR, L1TD1, LGALS3, MKRN1, NANOG, PDE1C, WNT11, CXCR4, FGF4, LINC00662,* and *RPS6KA5*) in NOA patients by real-time PCR. *CCND1, HMGA2, PDE1C,* and *LINC00662* were downregulated (*p* < 0.05), and *LGALS3, NANOG, WNT11, CXCR4* and *RPS6KA5* were upregulated (*p* < 0.05) in the male gonad tissue from an azoospermic patient with a mutation in the *ESX1* gene in comparison to an azoospermic patient without mutation in the *ESX1* gene as well as to control samples with normal spermatogenesis (Fig. 6). The *KDR, MKRN1,* and *FGF4* genes showed no significant changes in NOA patient with mutations in the *ESX1* gene compared to the other samples (Fig. 6).

## Discussion

The genetic causes of nonobstructive azoospermia are not yet well known; therefore, there is still a need to study the human genome to identify the gene variants that could be responsible for NOA. In recent years, due to the development of new NGS and genome editing technologies, new variants of genes that are potentially responsible for NOA have been identified. One such finding is the *ESX1* gene, of which novel, ultrarare variants (c.1040C > G, c.1042C > G) manifesting as a compound mutation were discovered by our group in whole-genome sequencing analysis in men with nonobstructive azoospermia at postmeiotic arrest^11^. Using Sanger sequencing, we confirmed the SNVs obtained from WGS analysis for these patients (Supplementary Fig. 1).

Commonly found in cytoskeletal proteins, HOX proteins and zinc-finger transcription factors, the polyproline-II helix binds to specific protein folds, including SH3, WW, GYF, EUV, profilin, and EVH1 domains^20–23^. Examination of several proteomic databases for ESX1-interacting proteins revealed one SH3-domain-containing candidate protein, Dlg4^24–26^. This cytoplasmic protein plays a role in neuronal signalling; however, this interaction has limited relevance here. It is possible that other ESX1 interactors could recruit other factors with affinity for the polyproline region of ESX1. The C-terminal end of the polyproline region of ESX1 has two types of proline-rich repeats (Fig. 1A): one containing a proline and leucine and one containing an arginine and valine. The compound mutation of p.Pro347Arg/p.Leu348Val substitutes the former type of repeat for the latter one. This substitution could potentially change the global structure of the region. These repeats could form molecular barriers, recruiting some binding partners and eliminating others. Therefore, mutation of the core residues of the mutated repeat likely shifts this putative molecular barrier for aligning molecules in three-dimensional space, which would be expected to destabilize or even eliminate functional complexes.

Summarizing the co-staining of ESX1 protein with each specific spermatogonia marker, such as: UTF1, MLH1 and c-KIT, we observed that ESX1 protein is presented in state 1 through 4 spermatogonia (Fig. 2). The co-staining of ESX1 with UTF1, which is a specific marker in state 0 and 1 undifferentiated spermatogonia, demonstrated the positive signal for both proteins in state 1. The spermatogonia in state 0 were positive only for UTF1 protein and negative for ESX1, which suggests that the expression of ESX1 protein starts in state 1 spermatogonia. The positive signals of ESX1 in MLH1- and c-KIT-positive cells were a strong evidence that ESX1 is also presented during further states of spermatogonia. The multiplex immunofluorescence staining of testis from the *Human Protein Atlas database* shows nuclear localization in state 1 and 4 spermatogonia as well as preleptotene spermatocytes for MLH1 protein and cytoplasmic localization in state 2–3 spermatogonia for c-KIT protein. The double staining of ESX1 and MLH1 proteins showed mostly positive signals for the same cells, suggesting that ESX1 is presented in state 1 and 4 spermatogonia, but not at the spermatocytes stage, where only MLH1-signal was strong. Whereas, the co-staining of ESX1 with c-KIT—a specific marker of spermatogonia in state 2–3, showed cytoplasmic staining for c-KIT and nucleus for ESX1 in the same cells, which indicates that ESX1 is also localized in state 2 and 3 spermatogonia.

It was previously noted that *Esx1* hemizygous mutant males were fertile, demonstrating that Esx1 is not essential for mouse spermatogenesis^27^. However, it was later proven that human ESX1 and mouse Esx1 proteins exhibit an overall high level of sequence divergence (34%), which concentrates mostly in the C-terminal domain implicated in the cell cycle control^28,29^. Because of these data, we decided to use a human germ cell model—TCam-2 cells, which do not express the *ESX1* gene. Activation of the *ESX1* gene in TCam-2 cells was successfully achieved at the mRNA and protein levels (Fig. 3), which allowed us to analyse by RNA-seq the general expression profile of TCam-2 cells with the activated *ESX1* gene in comparison to controls—TCam-2 wild type and negative control (TCam-2 cells with nonspecific gRNAs for the human genome) (Fig. 4). We selected over 50 genes connected with spermatogenesis and/or characterized by high expression levels in the testis that were differentially expressed in *ESX1*-activated TCam-2 cells compared to both controls. Using STRING gene database, we checked *ESX1* gene interactions, and we found that *ESX1* gene directly interacts with *ELF3* and *NANOG.* It also interacts indirectly with *CLDN4, VIM, CCND1, FGF4, SQSTM1, LGALS3, FGF13, CXCR4, KDR, PTBRB, SPP1, L1TD1, ZSCAN10, HMGA2, TIMP3, OGFBP5, ADAMTS6;* due to their interaction with the aforementioned *ELF3* and *NANOG* (Supplementary Fig. 3D). Interestingly, upon observation of RNA sequencing results, we noticed that the genes taking part in the mitotic and proliferation processes, or responsible for maintaining the spermatogonia population (*NANOG, FGF4, CXCR4, FGF13, VIM, L1TD1, HMGA2, ZSCAN10, ADAMTS9*) were downregulated in the *ESX1*-activated TCam-2 cells. On the other hand, genes participating in the differentiation or occurring at later stages of spermatogenesis (*ELF3, SPP1, TIMP3, KDR, WNT11, IGFBP5, PTPRB, SQSTM1, CLDN4, CCND1, CX3CL1, LGALS3*) showed higher expression in TCam-2 cells with *ESX1* activation in comparison to both applied controls. These results may suggest that *ESX1* gene regulates the genes participating in the differentiation process and preparing the spermatogonia to transit into the next stage of spermatogenesis. As for the remaining of the genes, which were either down or upregulated by the activation of *ESX1* gene but had no shown interaction in the STRING gene database, further studies should be made in order to test their possible interaction with *ESX1* gene. Thus we can conclude that *ESX1* gene may take part in the promotion of cell differentiation in spermatocytes and/or regulate the balance between cell proliferation and differentiation, through direct interaction with *NANOG*, involved in the proliferation process, and *ELF3*, involved in the differentiation of the cells. Functional studies are required to justify this hypothesis. We verified 13 key genes by real-time PCR due to their involvement in the cell cycle, proliferation and apoptosis and with high expression in the testes *(CCND1, HMGA2, KDR, L1TD1, LGALS3, MKRN1, NANOG, PDE1C, WNT11, CXCR4, FGF4, LINC00662,* and *RPS6KA5*) (Fig. 5). These genes were differentially expressed in cells with the activated *ESX1* gene compared to controls applied, and except for the *LINC00662* gene, we confirmed the results obtained from RNA-seq analysis. Therefore, as expected, *CCND1, KDR, LGALS3, PDE1C,* and *WNT11* were upregulated (*p* < 0.05), and *HMGA2, L1TD1, MKRN1, NANOG, CXCR4, FGF4,* and *RPS6KA5* were downregulated (*p* < 0.05) in TCam-2 cells with *ESX1* activation compared to controls (Fig. 5).

However, it is worth highlighting the results obtained in the male gonad tissues from a patient with azoospermia with a detected mutation in the *ESX1* gene, where we noted an inverse correlation. Hence, the genes that were downregulated (*NANOG, CXCR4* and *RPS6KA5*) in *ESX1*-activated TCam-2 cells in comparison to the control cells (Figs. 4, 5) were characterized by increasing expression in the patient with mutation in the *ESX1* gene in comparison to the control samples (Fig. 6). Similar to genes that were upregulated (*CCND1, PDE1C,* and *LINC00662*) in the *ESX1*-activated cells, a decreased expression of these genes was observed in the patient with *ESX*1 mutation in comparison to men with normal spermatogenesis and NOA patient without *ESX1* mutation.

Ultimately, 6 genes, *NANOG, CXCR4, RPS6KA5, CCND1, PDE1C,* and *LINC00662* were identified as participants in cell proliferation and differentiation, and together with the analysis of their expression levels, provided an interesting interpretation of the *ESX1* gene as promoting the process of cell differentiation in spermatocytes. The *NANOG* gene is a homeobox transcription factor and plays a key role in the pluripotency process. Normally, the *NANOG* gene is expressed in pluripotent cell lines, including embryonic stem (ES) and embryonic germ (EG) cells, and loss of NANOG causes a tendency for ES cells to differentiate^30^. In our study, activation of the *ESX1* gene led to the downregulation of *NANOG* gene expression in TCam-2 cells (Figs. 4, 5), whereas the mutation (with compound variants) in the *ESX1* gene in azoospermic humans was characterized by increased *NANOG* gene expression (Fig. 6). It can be suggested that the *ESX1* gene impacts *NANOG* gene expression, causing its decrease, and dividing cells may undergo differentiation towards spermatocytes. In turn, when there is a mutation in the *ESX1* gene, this process may be disturbed by maintaining increased *NANOG* gene expression, and thus, the balance between the division and differentiation of gametogenic cells may be disturbed. Another interesting gene that was also highlighted *CCND1*, which is responsible for controlling the cell cycle at the G1/S phase. In 293T cells, it was shown that the truncated CCND1 isoform did indeed promote cell proliferation and accelerated cell cycle progression^31^. In our case, the balance between the division and differentiation of gametogenic cells may also be destabilized since the mutation in the *ESX1* gene affects the downregulation of the *CCND1* gene (Fig. 6). The same applies to the *RPS6KA5* proliferation-promoting gene^32^, which in our study showed increased expression levels in the patient with a mutation in the *ESX1* gene and decreased expression in TCam-2 cells with an activated *ESX1* gene. Here, it also seems that under normal conditions, the *ESX1* gene potentially regulates the expression level of the *RPS6KA5* gene and consequently inhibits cell proliferation, leading to the differentiation process. Our hypothesis regarding the role of the *ESX1* gene in the promotion of cell differentiation in spermatocytes may also be supported by its influence on the *PDE1C* gene, which is expressed in germ cells from early meiotic prophase through the meiotic and postmeiotic stages^33^. In our studies, the *PDE1C* gene was characterized by increased expression levels in *ESX1*-activated TCam-2 cells (Figs. 4, 5), whereas in the NOA patient with *ESX1* gene mutation, *PDEC1* gene expression was decreased (Fig. 6). In turn, the *CXCR4* gene, which is a crucial receptor for spermatogonial stem cell maintenance^34^, was characterized in our studies by decreased expression in TCam-2 cells with an activated *ESX1* gene (Figs. 4, 5), while in the patient with *ESX1* gene mutation, upregulation of the *CXCR4* gene was observed (Fig. 6). This finding also suggests that the *ESX1* gene may regulate the process of spermatogonia proliferation and directs cells to differentiate into spermatocytes. The last selected gene is *LINC00662*, which is a spermatogenesis-associated lncRNA (long noncoding RNA); however, its function in spermatogenesis is still not clarified^35^. It was previously reported that *LINC00662* was upregulated in at least 14 tumours and promoted the proliferation of cancer cells^36^; however, its mechanism remains under study, and it is impossible to base our results to this information.

We compared our results to the most recently published information about the role of the *ESX1* gene in spermatogenesis, which showed that its protein product may participate in the regulation of cell cycle progression during human spermatogenesis^12^. A Chinese group observed that a mutation at position p.Pro365Arg/p.Leu366Val in ESX1 failed to inhibit cyclin A degradation and could cause cell cycle arrest^12^. The authors suggested that because cyclins also take part in the proliferation of spermatogonia, the discovered p.P365R mutation may negatively affect spermatogonia proliferation and cause spermatogenesis defects, demonstrating the SCO phenotype in the NOA patient harbouring the p.P365R mutation^12^. Admittedly, in our results, we did not observe any changes in cyclin A expression, while we documented a significant upregulation of cyclin D1 (*CCND1*) in *ESX1*-activated TCam-2 cells in comparison to controls (Fig. 4), whereas in the patient with *ESX1* mutation, the *CCND1* gene was significantly downregulated when compared to the controls (Fig. 6). Moreover, similar to the results reported by the Chinese group, in our previous studies, we identified the compound mutation p.Pro347Arg/p.Leu348Val in 2 patients with azoospermia^11^, which also affected the proline-rich repeat region, albeit a few amino acids upstream of the mutation discovered by the Chinese group^12^. However, in the case of our patients, they were characterized by postmeiotic arrest^11^, and together with the results presented in this manuscript, indicated the most likely hypothesis that the *ESX1* gene may take part in the promotion of cell differentiation in spermatocytes and/or more probably regulate the balance between cell proliferation and differentiation. Functional studies are still needed to justify this hypothesis.

To summarize our results, protein product of the *ESX1* gene is localized in the nucleus of state 1–4 spermatogonia and maybe involved in the regulation of cell differentiation into spermatocytes and/or more probably regulate the balance between cell proliferation and differentiation.

## Materials and methods

### Subjects

In this study, we used blood samples from 2 out of 39 NOA patients (marked as 10L and 33P). They were characterized by the compound mutation p.Pro347Arg/p.Leu348Val in the *ESX1* gene, detected in our previous research using whole-genome sequencing (WGS)^11^. Moreover, for the real-time PCR verification we used RNA extracted from the testicular tissue of a NOA patient with postmeiotic arrest without mutation in *ESX1* (marked as 2L) and a patient with NOA with compound mutation p.Pro347Arg/p.Leu348Val in the *ESX1* gene (marked as 10L)^11^. All the details concerning patients characterization were previously described by Malcher et al.^11^. As control sample, we used commercial RNA obtained from normal testicular tissue with preserved spermatogenesis pooled from 39 Caucasians aged between 16 and 64 years (Clontech Laboratories, Mountain View, CA, USA); whereas for the immunofluorescence staining we used the commercial human testis paraffin sections (Zyagen, San Diego, CA, USA). This study was approved by the Local Bioethics Committee of Poznan University of Medical Sciences (Permission No. 1003/18), and all participants provided informed consent. All experiments were performed in accordance with relevant guidelines and regulations.

### Extraction and sequencing of DNA from patient blood samples

Blood samples from 2 NOA patients with postmeiotic arrest caused by the compound mutation p.Pro347Arg/p.Leu348Val] in the *ESX1* gene, which was identified by WGS analysis^11^, were used to extract DNA. We performed Sanger sequencing of the *ESX1* gene for both studied patients to confirm the previously identified variants. The reaction mixture for sequencing contained 15–30 ng of DNA, 1 µl of primer for the *ESX1* gene (20 µM), 2 µl of BigDye (5x) buffer, and BigDye Terminator v3.1 (Applied Biosystems Life Technologies, Carlsbad, CA, USA) in a final volume of 20 µl. The primer pairs used for the sequencing reaction are listed in Supplementary Table 2. The products were separated on an ABI Prism 310 (Applied Biosystems, Life Technologies, Carlsbad, CA, USA). Changes in the patients’ sequenced *ESX1* fragments were identified with respect to the reference DNA (made available in the NCBI database) using the program CLC Workbench 6.0. The *ESX1* sequence of each patient was compared to the NCBI Reference Sequence GRCh37.p13: NC_000023.10 103,494,719.103499614.

### Structural protein modelling

The sequence of human ESX1 (Q8N693) was obtained from UniProt^18^, and residues 244–378 were searched in software representing iterative improvements of AlphaFold2, ColabFold2 AlphaFold2 and ColabFold_AlphaFold2_advanced_beta without relaxation^17,19^. Alignments were performed using LSQ-based alignment in Coot over the mainchain atoms^37^. Structure-based figures were made in PyMol (Version 2.0, Schrödinger, LLC).

### TCam-2 cell culture

TCam-2 cells were gifted by Dr Sohei Kitazawa (Ehime University Graduate School of Medicine, Japan) and were cultured in RPMI1640 medium supplemented with L-glutamine (Gibco, Thermo Fisher Scientific, MT, USA), 10% Foetal calf serum (GE Healthcare HyClone, Logan, Utah, USA), 1% penicillin/streptomycin (Lonza, Basel, Switzerland) and 2 mM glutamine (Lonza, Basel, Switzerland) in standard conditions at 37 °C, 5% CO2, and 95% humidity.

### Plasmid preparation and transfection

Three gRNAs were designed for the *ESX1* gene using the CRISPOR tool (http://crispor.tefor.net/). Candidate sequences for CRISPRa design were selected from specific regions of the genomic sequence relative to the gene's transcription start site (TSS) at positions from − 300 to 0 bases from the TSS region. The gRNA sequences are listed in the 5′-3′ direction: gRNA_1-AAGGCACCGTACCGCTATAT; gRNA_2-GACACGAATGCGTCCCCCGG; and gRNA_3-AAGGCACCGTACCGCTATAT. The selected gRNAs were cloned into the vector px335_G2P, which also includes GFP sequence. We performed co-transfection with 2.5 µg of DNA mix, which consisted of the 3 different px335_G2P vectors with cloned gRNAs specific for the *ESX1* gene and the PB-TRE-dCas9-VPR vector, using 3.75 µl of Lipofectamine 3000 in TCam-2 cells (passages 35–37). As a negative control we used px335_G2P vector with nonspecific gRNAs for the human genome. The vector pX335_G2P was a kind gift from Boris Greber (Max Planck Institute for Molecular Biomedicine, Münster) to the Dept. Herrmann at the Max Planck Institute for Molecular Genetics in Berlin, and PB-TRE-dCas9-VPR (Addgene #63,800, Addgene, Watertown, MA, USA) was purchased by the Max Planck Institute for Molecular Genetics in Berlin (Dept. Herrmann), which has the right to use the construct. The cells were selected with puro and doxycycline for 72 h. After 72 h, the cells were collected, and *ESX1* gene expression was analysed. The experiment was done in 3 biological replicates. The transfection efficiency of the modified TCam-2 cells was confirmed on a Juli FL fluorescence microscope (NanoEnTek, Seoul, South Korea) and the percentage of positively transfected cells (GFP-positive cells) was counted with multi-point tool using ImageJ2^38^.

### RNA, DNA and protein extraction

The cell or the testicular samples were lysed in 350 µl of RLT buffer with β-mercaptoethanol, and RNA, DNA and protein were extracted according to the manufacturer’s protocol of the AllPrep DNA/RNA/Protein Mini Kit (Qiagen, Hilden, Germany). The RNA samples were further purified according to the manufacturer’s protocol of the Turbo DNA-free Kit (Thermo Fisher Scientific, MT, USA). At the end of the procedure, the protein was dissolved in a buffer consisting of 8 M urea, 50 mM Tris–HCl, pH 8.0, 1% SDS (1:1) and a protease inhibitor cocktail (Roche, Basel, Switzerland).

### Real-time PCR

The cDNA was synthesized from 1 μg of total RNA using iScript™ Reverse Transcription Supermix (Bio-Rad Laboratories, Hercules, CA, USA) in a 20 μL reaction volume in a C1000 Touch thermocycler (Bio-Rad Laboratories, Hercules, CA, USA). Real-time PCR was performed using specific primers for the studied genes (detailed in Supplementary Table 2) with SsoAdvanced ™ SYBR ® Green Supermix (Bio-Rad Laboratories, Hercules, CA, USA) according to the manufacturer’s protocol. The threshold cycle (Ct) values of each transcript studied were analysed with a CFX384 Touch™ Real-time PCR detection system (Bio-Rad Laboratories, Hercules, CA, USA) using standard cycling parameters. All TCam2 cell samples—WT (n = 3 biological replicates), NC (n = 3 biological replicates), ESX1 (n = 3 biological replicates) as well as the standard curve were run in duplicate. The relative expression level of each studied transcript was normalized with reference to two housekeeping genes (*β-actin* and *GAPDH*) according to CFX Maestro software (Bio-Rad Laboratories, Hercules, CA, USA).

### Western blot

The protein extract from TCam-2 cells—WT (n = 3 biological replicates), NC (n = 2 biological replicates) and ESX1 (n = 3 biological replicates), was isolated at the opportunity of RNA extraction according to the manufacturer's protocol of Allprep DNA/RNA/protein Mini Kit (Qiagen, Hilden, Germany). At the end, the proteins were dissolved in 8 M urea, 50 mM Tris–HCl, pH 8.0 with 1% SDS (1:1) containing protease inhibitor cocktail (Roche, Basel, Switzerland). The total protein concentration was determined using the Lowry method. The 50 μg of protein was separated on 4–20% Mini-PROTEAN® TGX Stain-Free™ Protein Gels (Bio-Rad Laboratories, Hercules, CA, USA), electrotransferred in mix condition (7′) using Trans-Blot® Turbo (Bio-Rad Laboratories, Hercules, CA, USA) to PVDF membrane (Bio-Rad Laboratories, Hercules, CA, USA). The membrane was blocked with blocking buffer containing non-fat milk (Bio-Rad Laboratories, Hercules, CA, USA). Immunodetection was performed using the following antibodies: primary antibodies- anti-ESX1 rabbit (Invitrogen, Thermo Fisher Scientific, MT, USA) 1:500—65 kDa; anti-HPRT rabbit (Abcam, Cambridge, UK) 1:100—24 kDa; secondary antibodies- anti-rabbit (Abcam, Cambridge, UK) 1:40,000. The detection of the target protein was achieved by incubating the membrane with Clarity™ ECL Western Blotting Substrate (Bio-Rad Laboratories, Hercules, CA, USA) and analysed with ChemiDoc™ XRS system (Bio-Rad Laboratories, Hercules, CA, USA). The quantity analysis of the bands (ESX1 protein normalized with reference to HPRT1) was analysed by Image Lab 6.1 tools (Supplementary Fig. 4).

### Immunofluorescence staining

*For tissue samples*: The formalin-fixed, paraffin-embedded tissue section presenting normal spermatogenesis was deparaffinized in xylen (2 × 10 min) and rehydrated 2 × 5 min in 100% ethanol, 96% ethanol, and 70% ethanol, respectively. The antigen retrieval was performed as follows: incubation in 2% NaBH4 for 30 min at room temperature, then another 30 min in 0.1% glycine at room temperature and in 0.01% sodium citrate at 95 °C for the next 30 min. After rinsing in 1xPBS, the sections were blocked with 10% goat serum (in 0.1% Triton-100 in 1xPBS) for 1 h and then incubated overnight with anti- ESX1 rabbit polyclonal antibody (Invitrogen, Thermo Fisher Scientific, MT, USA) 1:100; anti- UTF1 mouse monoclonal antibody (Invitrogen, Thermo Fisher Scientific, MT, USA) 1:50; anti- MLH1 mouse monoclonal antibody (Abcam, Cambridge, UK) 1:30; anti-c-Kit monoclonal antibody 1:50 (diluted in 0.1% Triton-100 in 1xPBS) at 4 °C. After rinsing 2 × 5 min with 1xPBS, the sections were incubated with anti-rabbit antibody and anti-mouse antibody (Abcam, Cambridge, UK) 1:400 (diluted in 1xPBS) for 1 h at room temperature. After this time, the tissues/cells were washed 3 times with 1xPBS, the DAPI was applied, and then covered with a coverslip. For tissue samples 4 different staining combinations were performed with 3 technical replicates for each staining. Images were acquired using a fluorescence microscope with a proper filter set: Leica DM5500, filters: DAPI/TxR/SpG/Triple; objectives: 10 × and 63 × with oil immersion; software: CytoVision.

*For cell samples*: First the cells were fixed with 4% paraformaldehyde and then the cell membranes were permeabilized with 0.1% Triton-100 in 1xPBS and then incubated for 15 min. After rinsing in 1xPBS, the sections were blocked with 10% goat serum (in 0.1% Triton-100 in 1xPBS) for 1 h and then incubated overnight with anti-ESX1 (Invitrogen, Thermo Fisher Scientific, MT, USA) 1:200 (diluted in 0.1% Triton-100 in 1xPBS) at 4 °C. After rising 2 × 5 min with 1xPBS, the sections were incubated with anti-rabbit antibody (Abcam, Cambridge, UK) 1:500 (diluted in 1xPBS) for 1 h at room temperature. After this time, the tissues/cells were washed 3 times with 1xPBS, the DAPI was applied, and then covered with a coverslip. For TCam-2 cell samples—immunostaining for ESX1 protein was performed in 2 biological replicates for each studied samples (WT, NC, ESX1). A Leica DMi8 with a proper filter set (DAPI/TxR/Triple) was used; objectives: 40x, scale bar: 50 μm; software: LASX. The percentage of ESX1-positive cells was counted with multi-point tool using ImageJ2^38^.

### RNA sequencing (RNA-seq)

Sequencing libraries were prepared from total RNA (300 ng) of studied TCam-2 cell samples: WT (n = 3 biological replicates), NC (n = 3 biological replicates) and ESX1 (n = 3 biological replicates), according to the library prep protocol for the TruSeq Stranded Total RNA with Ribo-Zero Human kit (Illumina), and ultrahigh-throughput sequencing with 40 million reads was performed using a NovaSeq6000 (Illumina) system. The paired-end sequences were 2 × 150 bases in length. The RNA-seq was performed commercially by a custom service. Bioinformatic and statistical analyses were performed as previously described^39^; the HISAT2 2.0.5 was used to align the sequenced reads to the GRCh38.p7 reference genome^40^. The featureCounts was used to count the aligned reads within adequate GENCODE v25 gene annotation regions^41,42^. The DESeq2 in the R/Bioconductor environment was used to normelize the read counts^43,44^. Expression data have been deposited in the NCBI Gene Expression Omnibus (GEO), Accession Number GSE227497. To select genes from the RNAseq data, the following criteria were set:

1. At least a twofold change in gene expression was detected
2. A *p* value < 0.05 was considered statistically significant
3. The level of gene expression in the testis was verified using NCBI Gene and/or EMBL-EBI (Illumina Body Map)
4. The level of gene expression in gametogenic cells was verified using Human Protein Atlas
5. The phenotypes, diseases and features associated with selected genes were determined using Ensembl
6. The function and association with infertility/spermatogenesis were determined using PubMed with the following keywords: "spermatogenesis" or "infertility" or “azoospermia” or “reproductive biology".

### STRING interactions

For analysis of possible interactions of the ESX1 protein with protein products of selected genes due to performed RNAseq analysis, STRING analysis was carried out (https://string-db.org/; version 11.5). The STRING database includes direct and indirect interactions between proteins, including computational predictions, knowledge transfer between organisms, and interactions aggregated from other databases^45^.

### Supplementary Information

Supplementary Information.

## Data Availability

Expression data have been deposited in the NCBI Gene Expression Omnibus (GEO), Accession Number GSE227497. All data included in this study are available upon request by contacting the corresponding author.
