# Supplementary material for: ESX1 gene as a potential candidate responsible for male infertility in nonobstructive azoospermia
Source: Sci Rep. 2023 Oct 2;13:16563. doi: 10.1038/s41598-023-43854-9 (PMC10545701; doi:10.1038/s41598-023-43854-9)
Supplement: Supplementary file 1 — Supplementary Information. [file 41598_2023_43854_MOESM1_ESM.pdf]

Supplementary Figure and Table legends:

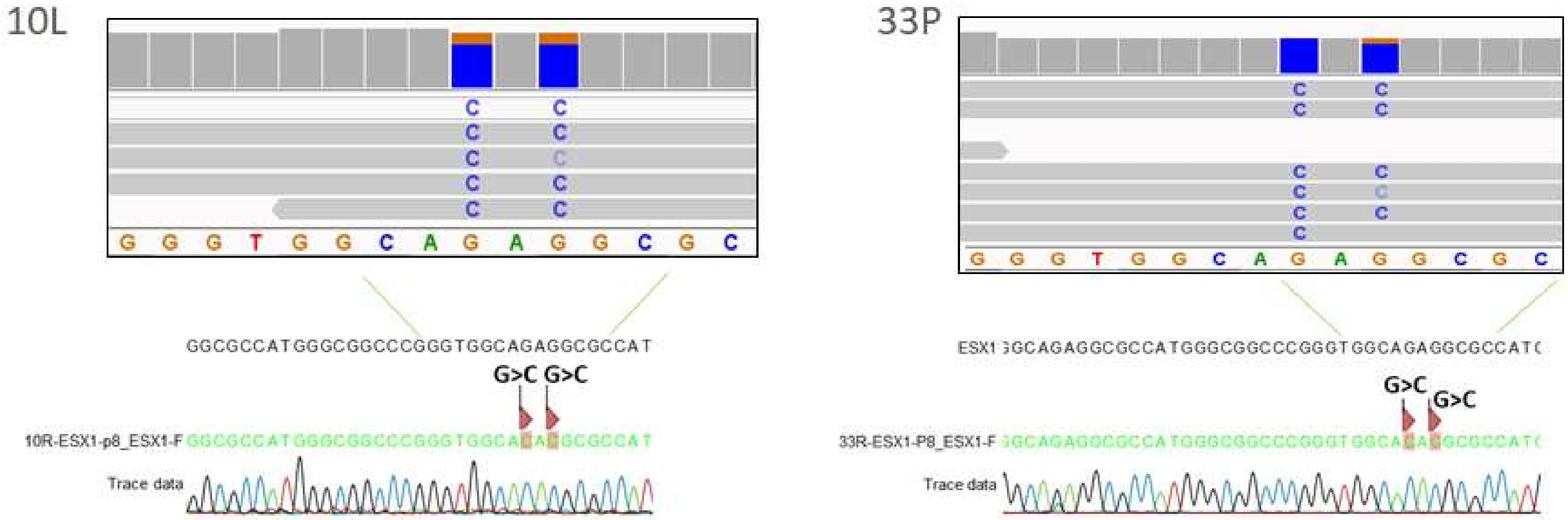

Supplementary Figure 1. SNVs (single nucleotide variants) in the *ESX1* gene identified from the bam file produced from WGS and Sanger sequencing analysis of samples from NOA patients previously identified by Malcher et al. (2022)[11].

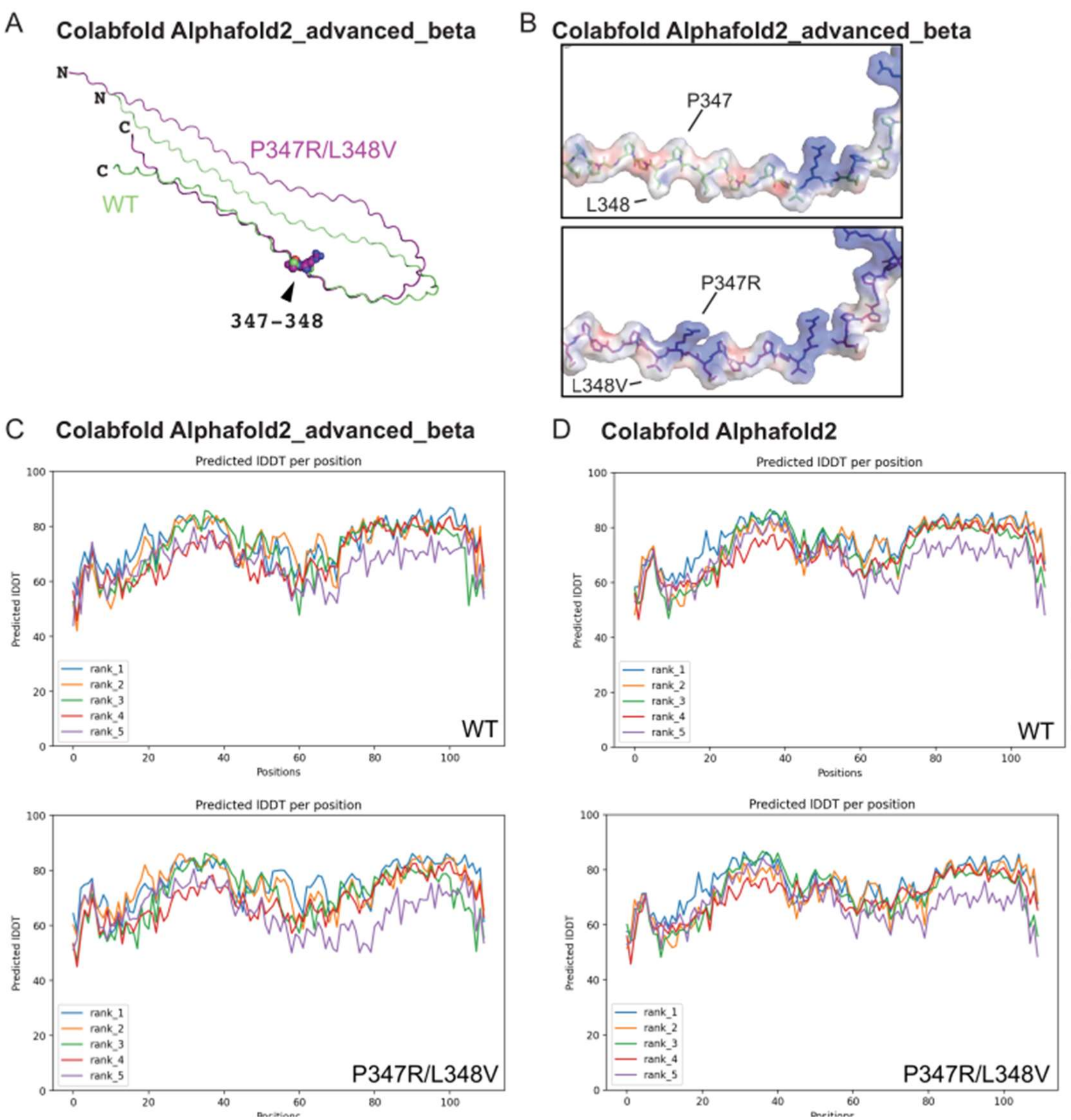

Supplementary Figure 2. Structural models of the ESX1 polyproline region as modelled by two iterations of AlphaFold. **A)** Alignment of ColabFold AlphaFold2-advanced beta-generated structures of the proline-rich regions of wild-type and P347R/L348V ESX1 variants shown as spheres. https://colab.research.google.com/github/sokrypton/ ColaFold/blob/main/beta/AlphaFold2_advanced_beta.ipynb. (Mirdita). **B)** Zoomed view of the surface representation of the region surrounding residues 347 and 348 modelled by ColabFold AlphaFold2_advanced beta. The surface is coloured by electrostatic potential as calculated in PyMOL; blue, positive; red, negative (~-30-30 eV). **C)** and **D)** Predicted LDDT scores per position for the top five generated models for each variant from **(C)** ColabFold AlphaFold1_advanced_beta and **(D)** ColabFold AlphaFold2. Residue positions are numbered sequentially, where position 1 is residue 244 and position 110 is residue 378. In this numbering scheme, the residues of interest are 79 and 80.

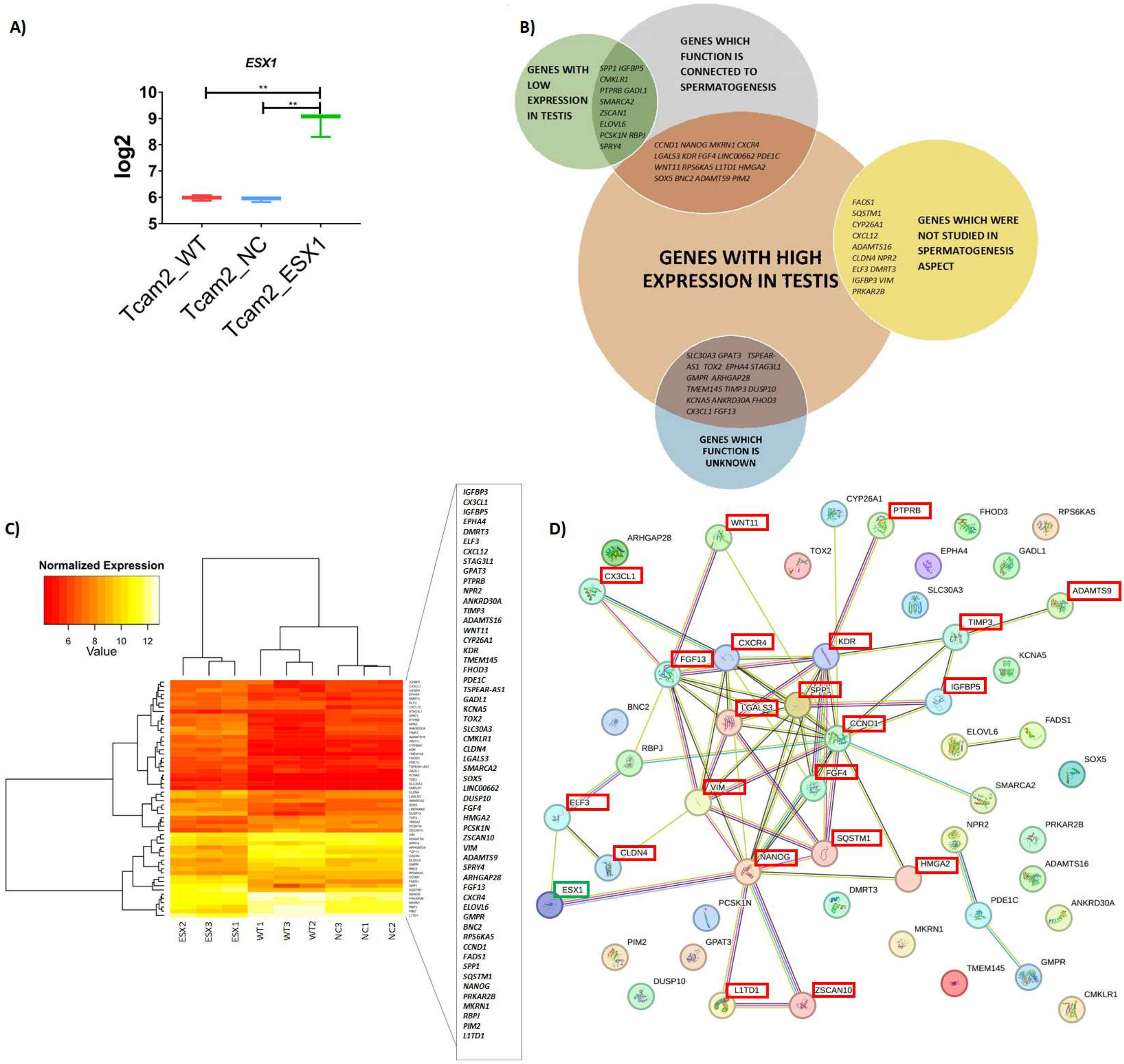

Supplementary Figure 3. RNA sequencing (RNA-seq) analysis revealed genes that were differentially expressed following activation of the *ESX1* gene. **A)** Gene expression level of *ESX1* in Tcam-2 cells analysed by RNA-seq. **B)** Venn diagram of the selected genes. **C)** Heatmap of the 54 selected differentially expressed genes ($p<0.05$) in *ESX1*-activated TCam-2 cells and controls. **D)** STRING gene analysis of potential interactions between ESX1 and protein products of genes selected from RNA-seq. The most important proteins are bracketed.

Legend: WT- wild type; NC- negative control with nonspecific gRNAs for the human genome; ESX1- cells with the activated *ESX1* gene using specific gRNAs for the *ESX1* sequence.

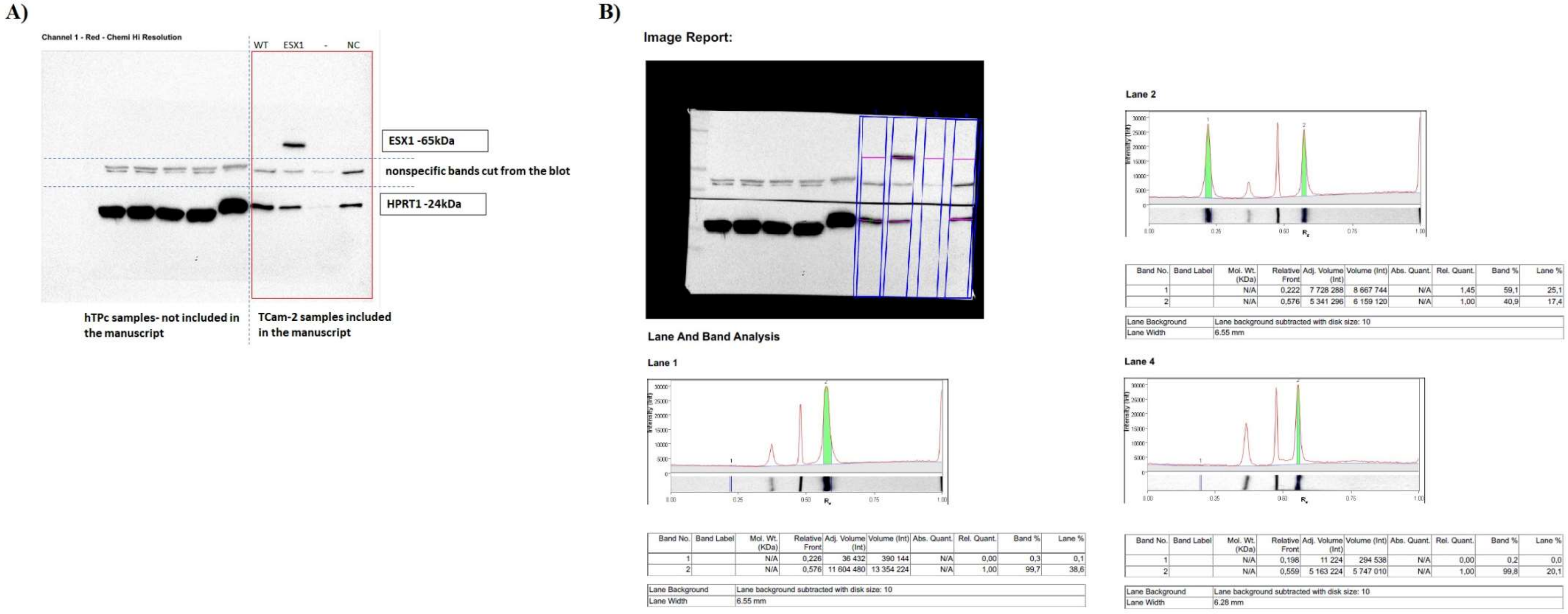

Supplementary Figure 4. Original blot used in the Figure 3 of the main manuscript. **A)** Image of the original blot. Exposure 300sec.; Chemi Hi resolution image. In the red frame are shown the protein samples included in the Figure 3; blue dashed lines show the cropped membrane; **B)** Report of the quantity analysis of the band**s** analyzed by Image Lab 6.1 tools.

Legend: hTPc- human primary testicular cells; TCam-2- testicular seminoma cell; WT- wild type; NC- negative control with nonspecific gRNAs for the human genome; ESX1- cells with the activated ESX1 gene using specific gRNAs for the ESX1 sequence.

Supplementary Table 1. Gene Ontology analysis (CPDB, ConsensusPathDB database) of the genes selected from RNA-seq.

**Enriched pathway-based sets**									
P-value	Q-value	Pathway	Source	External_id	Members_input_overlap	Members_input_overlap_geneids	Size	Effective_size	Candidates
9.96063618671e-05	0.00962873297616	Signal Transduction	Reactome	R-HSA-162582	SQSTM1; CXCL12; RPS6KA5; ARHGAP28; CCND1; RBPJ; VIM; MKRN1; PDE1C; PRKAR2B; SPP1; KDR; CXCR4; CX3CL1; FGF4; CMKLR1; DUSP10; WNT11; CYP26A1	7431; 595; 3516; 6696; 2249; 5577; 11221; 7481; 9252; 3791; 1240; 6387; 23608; 6376; 8878; 1592; 7852; 5137; 79822	2432	2431	contained

0.000112867483007	0.00962873297616	Neovascularisation processes	Wikipathways	WP4331	CXCL12; KDR; CXCR4	7852; 6387; 3791	29	29	3 (10.3%)
0.000138211478127	0.00962873297616	activation of camp-dependent protein kinase pka	BioCarta	gspathway	PRKAR2B; CXCL12; CXCR4	6387; 5577; 7852	31	31	3 (9.7%)
0.000217044912413	0.00987459859178	The influence of laminopathies on Wnt signaling	Wikipathways	WP4844	CCND1; SPP1; HMGA2	595; 8091; 6696	36	36	3 (8.3%)
0.000321511961248	0.00987459859178	Vitamin D Receptor Pathway	Wikipathways	WP2877	CCND1; TIMP3; SPP1; DUSP10; IGFBP5	3488; 595; 11221; 6696; 7078	184	184	5 (2.7%)
0.000344223775894	0.00987459859178	chrebp regulation by carbohydra	BioCarta	chrebppathway	PRKAR2B; CXCL12; CXCR4	6387; 5577; 7852	42	42	3 (7.1%)

		tes and camp							
0.000369196520093	0.00987459859178	Genes controlling nephrogenesis	Wikipathways	WP4823	CXCL12; KDR; CXCR4	7852; 6387; 3791	43	43	3 (7.0%)
0.000451028947287	0.00987459859178	activation of csk by camp-dependent protein kinase inhibits signaling through the t cell receptor	BioCarta	cskpathway	PRKAR2B; CXCL12; CXCR4	6387; 5577; 7852	46	46	3 (6.5%)
0.000527566937861	0.00987459859178	Proteoglycans in cancer - *Homo sapiens* (human)	KEGG	path:hsa05205	CCND1; NANOG; TIMP3; WNT11; KDR	595; 3791; 7481; 7078; 79923	205	205	5 (2.4%)

0.000565920995409	0.00987459859178	cxcr4 signaling pathway	BioCarta	cxcr4pathway	CXCL12; CXCR4	7852; 6387	11	11	2 (18.2%)
0.000565920995409	0.00987459859178	pertussis toxin-insensitive ccr5 signaling in macrophage	BioCarta	ccr5pathway	CXCL12; CXCR4	7852; 6387	11	11	2 (18.2%)
0.000566962598571	0.00987459859178	Spinal Cord Injury	Wikipathways	WP2431	LGALS3; CCND1; EPHA4; VIM	3958; 7431; 2043; 595	117	117	4 (3.4%)
0.000677692174243	0.0102261399688	Omega-9 FA synthesis	Wikipathways	WP4724	ELOVL6; FADS1	79071; 3992	12	12	2 (16.7%)
0.000685004591214	0.0102261399688	Cardiac Progenitor Differentiation	Wikipathways	WP2406	NANOG; KDR; CXCR4	3791; 7852; 79923	53	53	3 (5.7%)
0.000799242820221	0.0104401093391	Mammary gland developme	Wikipathways	WP2814	CCND1; VIM	595; 7431	13	13	2 (15.4%)

		nt pathway - Puberty (Stage 2 of 4)							
0.00079924 2820221	0.01044010 93391	p53 signaling pathway	BioCarta	p53pathway	CCND1; TIMP3	7078; 595	13	13	2 (15.4%)
0.00108343 935107	0.01248523 83097	Chemokine receptors bind chemokine s	Reactom e	R-HSA-380108	CX3CL1; CXCL12; CXCR4	6387; 7852; 6376	62	62	3 (4.8%)
0.00113502 166451	0.01248523 83097	Endochond ral Ossificatio n	Wikipat hways	WP474	SPP1; TIMP3; SOX5	6660; 7078; 6696	63	63	3 (4.8%)
0.00113502 166451	0.01248523 83097	Endochond ral Ossificatio n with Skeletal Dysplasias	Wikipat hways	WP4808	SPP1; TIMP3; SOX5	6660; 7078; 6696	63	63	3 (4.8%)

0.00155151658289	0.0154963406595	Mammary gland development pathway - Embryonic development (Stage 1 of 4)	Wikipathways	WP2813	CCND1; CLDN4	595; 1364	18	18	2 (11.1%)
0.00155704858302	0.0154963406595	Pathways in cancer - *Homo sapiens* (human)	KEGG	path:hsa05200	FGF4; PIM2; RPS6KA5; CCND1; CXCR4; CXCL12; WNT11	2249; 6387; 595; 11040; 7481; 9252; 7852	531	531	7 (1.3%)
0.00191872286582	0.0174353512589	Angiogenesis	Wikipathways	WP1539	TIMP3; KDR	3791; 7078	20	20	2 (10.0%)
0.00191872286582	0.0174353512589	sprouty regulation of tyrosine kinase signals	BioCarta	sprypathway	PTPRB; SPRY4	5787; 81848	20	20	2 (10.0%)

0.002323084703	0.0194209881171	Extracellular vesicles in the crosstalk of cardiac cells	Wikipathways	WP4300	SPP1; KDR	3791; 6696	22	22	2 (9.1%)
0.002323084703	0.0194209881171	cystic fibrosis transmembrane conductance regulator (cftr) and beta 2 adrenergic receptor (b2ar) pathway	BioCarta	cftrpathway	PRKAR2B; CXCL12	6387; 5577	22	22	2 (9.1%)
0.00253904897333	0.0204100475164	FGF23 signaling in hypophosphatemic rickets and	Wikipathways	WP4790	CCND1; SPP1	595; 6696	23	23	2 (8.7%)

		related disorders							
0.00277589012156	0.0214874457558	Pathways in clear cell renal cell carcinoma	Wikipathways	WP4018	SQSTM1; TOX2; KDR	3791; 8878; 84969	86	86	3 (3.5%)
0.00329173658675	0.0240889386172	MicroRNAs in cancer - *Homo sapiens* (human)	KEGG	path:hsa05206	CCND1; TIMP3; HMGA2; RPS6KA5; VIM	9252; 595; 7431; 8091; 7078	310	310	5 (1.6%)
0.0034934097949	0.0240889386172	Biosynthesis of unsaturated fatty acids - *Homo sapiens* (human)	KEGG	path:hsa01040	FADS1; ELOVL6	3992; 79071	27	27	2 (7.4%)
0.0034934097949	0.0240889386172	transcription factor creb and its extracellular signals	BioCarta	crebpathway	PRKAR2B; RPS6KA5	9252; 5577	27	27	2 (7.4%)

0.00357300046475	0.0240889386172	G alpha (i) signalling events	Reactome	R-HSA-418594	PDE1C; CX3CL1; CXCL12; PRKAR2B; CXCR4	5137; 5577; 7852; 6387; 6376	316	316	5 (1.6%)
0.00375433184126	0.0245204798382	Transcriptional regulation of pluripotent stem cells	Reactome	R-HSA-452723	ZSCAN10; NANOG	84891; 79923	28	28	2 (7.1%)
0.0042486780363	0.0260880425562	Viral protein interaction with cytokine and cytokine receptor - *Homo sapiens* (human)	KEGG	path:hsa04061	CX3CL1; CXCL12; CXCR4	7852; 6376; 6387	100	100	3 (3.0%)

0.00430256623035	0.0260880425562		BioCarta	barrestinpathway	CXCL12; CXCR4	6387; 7852	30	30	2 (6.7%)
0.00436881095439	0.0260880425562	Wnt signaling pathway and pluripotency	Wikipathways	WP399	CCND1; NANOG; WNT11	595; 7481; 79923	101	101	3 (3.0%)
0.00461523727805	0.0261818225546	Degradation of the extracellular matrix	Reactome	R-HSA-1474228	ADAMTS9; SPP1; ADAMTS16	6696; 56999; 170690	103	103	3 (2.9%)
0.00481330190405	0.0261818225546	PI3K-Akt signaling pathway	Wikipathways	WP4172	CCND1; FGF13; FGF4; SPP1; KDR	2249; 595; 2258; 3791; 6696	340	339	5 (1.5%)
0.00488560325182	0.0261818225546	Syndecan-4-mediated signaling events	PID	syndecan_4_pathway	CXCL12; CXCR4	6387; 7852	32	32	2 (6.2%)
0.00488560325182	0.0261818225546	role of -arrestins in the activation	BioCarta	barrmapkpathway	CXCL12; CXCR4	6387; 7852	32	32	2 (6.2%)

		and targeting of map kinases							
0.00513301606701	0.0268200089501	Purine nucleotides nucleosides metabolism	INOH	None	PDE1C; NPR2; GMPR	5137; 2766; 4882	107	107	3 (2.8%)
0.00612070277565	0.0294983738577	Leukocyte transendothelial migration - *Homo sapiens* (human)	KEGG	path:hsa04670	CXCL12; CLDN4; CXCR4	7852; 6387; 1364	114	114	3 (2.6%)
0.00615425921148	0.0294983738577	roles of arrestin dependent recruitment of src kinases in gpcr signaling	BioCarta	barrestinsrcpathway	CXCL12; CXCR4	6387; 7852	36	36	2 (5.6%)

0.0061564142032	0.0294983738577	Human cytomegalovirus infection - *Homo sapiens* (human)	KEGG	path:hsa05163	CCND1; CX3CL1; CXCL12; CXCR4	595; 7852; 6387; 6376	225	225	4 (1.8%)
0.00642232052568	0.0294983738577	ESC pluripotency Pathways	Wikipathways	WP3931	FGF13; FGF4; WNT11	2249; 2258; 7481	116	116	3 (2.6%)
0.0064924650596	0.0294983738577	Calmodulin induced events	Reactome	R-HSA-111933	PDE1C; PRKAR2B	5137; 5577	37	37	2 (5.4%)
0.0064924650596	0.0294983738577	CaM pathway	Reactome	R-HSA-111997	PDE1C; PRKAR2B	5137; 5577	37	37	2 (5.4%)
0.00673269440301	0.0299390027708	Osteoblast differentiation	Wikipathways	WP4787	RBPJ; FGF4; WNT11	2249; 3516; 7481	118	118	3 (2.5%)
0.00705188844881	0.0299497963331	Hippo-Merlin Signaling Dysregulation	Wikipathways	WP4541	CCND1; PRKAR2B; KDR	3791; 595; 5577	120	120	3 (2.5%)

0.00719373588336	0.0299497963331	Ca-dependent events	Reactome	R-HSA-111996	PDE1C; PRKAR2B	5137; 5577	39	39	2 (5.1%)
0.00719373588336	0.0299497963331	O-glycosylation of TSR domain-containing proteins	Reactome	R-HSA-5173214	ADAMTS9; ADAMTS16	56999; 170690	39	39	2 (5.1%)
0.00753080841776	0.0299497963331	Signaling by GPCR	Reactome	R-HSA-372790	PDE1C; PRKAR2B; CXCR4; CX3CL1; CXCL12; CMKLR1; WNT11	1240; 6387; 6376; 7481; 5577; 7852; 5137	706	706	7 (1.0%)
0.00755669016025	0.0299497963331	Bladder cancer	Wikipathways	WP2828	CCND1; RPS6KA5	595; 9252	40	40	2 (5.0%)
0.00759492442896	0.0299497963331	Calcium signaling pathway - *Homo*	KEGG	path:hsa04020	PDE1C; FGF4; KDR; CXCR4	5137; 7852; 3791; 2249	240	239	4 (1.7%)

		sapiens (human)							
0.00792778364681	0.0306834589293	Bladder cancer - *Homo sapiens* (human)	KEGG	path:hsa05219	CCND1; RPS6KA5	9252; 595	41	41	2 (4.9%)
0.00830696164035	0.0307670355663	actions of nitric oxide in the heart	BioCarta	no1pathway	PRKAR2B; KDR	3791; 5577	42	42	2 (4.8%)
0.00830696164035	0.0307670355663	DAG and IP3 signaling	Reactome	R-HSA-1489509	PDE1C; PRKAR2B	5137; 5577	42	42	2 (4.8%)
0.00839100969989	0.0307670355663	MAPK Signaling Pathway	Wikipathways	WP382	FGF13; FGF4; RPS6KA5; DUSP10	2249; 2258; 9252; 11221	246	246	4 (1.6%)
0.00859901512615	0.0309861062304	Purine metabolism - *Homo sapiens* (human)	KEGG	path:hsa00230	PDE1C; NPR2; GMPR	5137; 2766; 4882	130	129	3 (2.3%)
0.00908935362063	0.0321978797748	Beta3 integrin	PID	integrin3_pathway	SPP1; KDR	3791; 6696	44	44	2 (4.5%)

		cell surface interactions							
0.00990343 359709	0.03318991 5543	Presenilin action in Notch and Wnt signaling	PID	ps1pathway	CCND1; RBPJ	595; 3516	46	46	2 (4.3%)
0.00990343 359709	0.03318991 5543	Translation inhibitors in chronically activated PDGFRA cells	Wikipat hways	WP4566	PIM2; RPS6KA5	9252; 11040	46	46	2 (4.3%)
**Enriched gene ontology-based sets**									
p-value	q-value	term_goid	term_ca tegory	term_level	term_name	members_input_ov erlap	members_input_ov erlap_geneids	size	effectiv e_size
2.80514083 413e-07	2.02797595 136e-05	GO:004222 1	b	2	response to chemical	595; 1240; 1364; 1592; 2043; 2249; 3488; 3516; 3741; 3791; 3958; 4882; 5577; 6376; 6387; 6660; 6696; 7078; 7431; 7481; 7852; 8091; 8878; 9252; 11221; 7781;	TIMP3; EPHA4; PRKAR2B; SPRY4; KCNA5; SLC30A3; SOX5; FGF4; NANOG; RBPJ; CXCR4; CX3CL1; CLDN4; KDR; NPR2; HMGA2; TMEM145; IGFBP5; LGALS3; CXCL12; DUSP10;	4693	4688

						79923; 81848; 284339	SQSTM1; RPS6KA5; CCND1; VIM; SPP1; CYP26A1; CMKLR1; WNT11		
4.31484244 97e-07	2.02797595 136e-05	GO:000960 5	b	2	response to external stimulus	595; 1240; 1592; 2043; 2249; 3516; 3741; 3791; 3958; 3992; 6376; 6387; 6696; 7431; 7481; 7852; 8091; 9252; 11040; 11221; 27344	CX3CL1; PCSK1N; KCNA5; PIM2; RPS6KA5; CCND1; WNT11; RBPJ; EPHA4; HMGA2; VIM; FADS1; CXCL12; SPP1; CYP26A1; CXCR4; LGALS3; FGF4; CMKLR1; DUSP10; KDR	2572	2570
1.10052887 859e-06	2.61269194 094e-05	GO:004886 9	b	2	cellular developmenta l process	595; 1240; 1364; 2043; 2249; 2258; 3488; 3516; 3791; 3958; 3992; 4882; 6376; 6387; 6595; 6660; 6696; 7431; 7481; 7852; 8091; 8878; 9252; 11221; 79923; 81848; 58524	DMRT3; EPHA4; LGALS3; SPRY4; SMARCA2; SOX5; FGF4; NANOG; RBPJ; CXCR4; CX3CL1; CLDN4; KDR; NPR2; HMGA2; FGF13; IGFBP5; FADS1; CXCL12; DUSP10; SQSTM1; RPS6KA5; CCND1; VIM; SPP1; CMKLR1; WNT11	4370	4365
1.26042200 558e-06	2.61269194 094e-05	GO:000715 4	b	2	cell communicatio n	595; 1240; 1592; 2043; 2249; 2258; 3488; 3516; 3741; 3791; 3958; 3992; 4882; 5137; 5577; 6376; 6387; 6696;	DMRT3; TIMP3; EPHA4; GPAT3; LGALS3; PRKAR2B; SPRY4; KCNA5; FGF4; NANOG; PDE1C; RBPJ;	6715	6710

						7078; 7431; 7481; 7852; 8091; 8878; 9252; 11040; 11221; 79923; 81848; 84803; 79822; 284339; 58524; 27344	CXCR4; CX3CL1; ARHGAP28; KDR; NPR2; PIM2; HMGA2; FGF13; TMEM145; IGFBP5; FADS1; CXCL12; DUSP10; SQSTM1; PCSK1N; RPS6KA5; CCND1; VIM; SPP1; CYP26A1; CMKLR1; WNT11		
1.38972975582e-06	2.61269194094e-05	GO:0007275	b	2	multicellular organism development	595; 1240; 1364; 1592; 2043; 2249; 2258; 3488; 3516; 3791; 3958; 4882; 5787; 6376; 6387; 6595; 6660; 6696; 7431; 7481; 7852; 8091; 170690; 9252; 11221; 79923; 81848; 54796; 56999; 58524	DMRT3; EPHA4; ADAMTS16; SMARCA2; SOX5; FGF4; NANOG; RBPJ; ADAMTS9; CXCR4; CX3CL1; CLDN4; KDR; PTPRB; NPR2; HMGA2; SPRY4; FGF13; IGFBP5; LGALS3; CXCL12; DUSP10; RPS6KA5; BNC2; CCND1; VIM; SPP1; CYP26A1; CMKLR1; WNT11	5363	5356
2.60917732782e-06	4.08771114693e-05	GO:0009653	b	2	anatomical structure morphogenesis	1364; 2043; 2249; 2258; 3488; 3516; 3791; 5787; 6387; 6660; 6696; 7481; 7852; 8091; 170690; 9252; 79923; 81848; 56999; 58524	ADAMTS16; CXCL12; NANOG; CXCR4; WNT11; RBPJ; EPHA4; HMGA2; SPRY4; RPS6KA5; DMRT3; SPP1; PTPRB; IGFBP5; FGF13; ADAMTS9;	2614	2610

							FGF4; CLDN4; KDR; SOX5		
3.94745319 272e-06	5.30086571 594e-05	GO:005078 9	b	2	regulation of biological process	595; 1240; 1364; 1592; 2043; 2249; 2258; 3488; 3516; 3741; 3791; 3958; 3992; 4882; 5137; 5577; 5787; 6376; 6387; 6595; 6660; 6696; 7078; 7431; 7481; 7852; 8091; 170690; 8878; 9252; 11040; 11221; 79071; 7781; 79923; 81848; 84803; 84891; 84969; 79822; 54796; 284339; 56999; 58524; 27344	DMRT3; TIMP3; EPHA4; GPAT3; LGALS3; PRKAR2B; ADAMTS16; SMARCA2; KCNA5; SLC30A3; SOX5; FGF4; NANOG; PDE1C; RBPJ; ADAMTS9; TOX2; CXCR4; CX3CL1; ARHGAP28; CLDN4; KDR; PTPRB; NPR2; PIM2; HMGA2; SPRY4; FGF13; TMEM145; IGFBP5; FADS1; CXCL12; DUSP10; SQSTM1; PCSK1N; RPS6KA5; BNC2; CCND1; VIM; ELOVL6; SPP1; CYP26A1; ZSCAN10; CMKLR1; WNT11	11854	11839
9.80795652 215e-06	9.88803064 213e-05	GO:007208 9	b	2	stem cell proliferation	2258; 6376; 6660; 8091; 79923	FGF13; CX3CL1; NANOG; HMGA2; SOX5	112	112
1.03707953 358e-05	9.88803064 213e-05	GO:004885 6	b	2	anatomical structure development	595; 1240; 1364; 1592; 2043; 2249; 2258; 3488; 3516; 3791; 3958; 4882; 5787; 6376; 6387; 6595; 6660; 6696;	DMRT3; EPHA4; ADAMTS16; SMARCA2; SOX5; FGF4; NANOG; RBPJ; ADAMTS9; CXCR4; CX3CL1; CLDN4;	5866	5857

						7431; 7481; 7852; 8091; 170690; 9252; 11221; 79923; 81848; 54796; 56999; 58524	KDR; PTPRB; NPR2; HMGA2; SPRY4; FGF13; IGFBP5; LGALS3; CXCL12; DUSP10; RPS6KA5; BNC2; CCND1; VIM; SPP1; CYP26A1; CMKLR1; WNT11		
1.05191815342e-05	9.88803064213e-05	GO:0007165	b	2	signal transduction	595; 1240; 1592; 2043; 2249; 2258; 3488; 3516; 3741; 3791; 3958; 4882; 5137; 5577; 6376; 6387; 6696; 7078; 7431; 7481; 7852; 8878; 9252; 11040; 11221; 79923; 81848; 84803; 79822; 284339; 27344	TIMP3; EPHA4; GPAT3; PRKAR2B; SPRY4; KCNA5; FGF4; NANOG; PDE1C; RBPJ; CXCR4; CX3CL1; ARHGAP28; KDR; NPR2; PIM2; FGF13; TMEM145; IGFBP5; LGALS3; CXCL12; DUSP10; SQSTM1; PCSK1N; RPS6KA5; CCND1; VIM; SPP1; CYP26A1; CMKLR1; WNT11	6210	6205
1.45081312622e-05	0.000123978576241	GO:0006928	b	2	movement of cell or subcellular component	1240; 1364; 2043; 2249; 2258; 3488; 3741; 3791; 3958; 6376; 6387; 7431; 7481; 7852; 9252; 11221; 56999	CX3CL1; KCNA5; CXCR4; CMKLR1; EPHA4; ADAMTS9; VIM; LGALS3; CXCL12; WNT11; IGFBP5; FGF13; FGF4; CLDN4; DUSP10; KDR; RPS6KA5	2164	2162

1.61378686431e-05	0.000126413304371	GO:0065008	b	2	regulation of biological quality	595; 1240; 1364; 1592; 2043; 2258; 3488; 3516; 3741; 3791; 4882; 5577; 6376; 6387; 6696; 7431; 7852; 170690; 8878; 11040; 79071; 7781; 79822; 27344	EPHA4; PRKAR2B; ADAMTS16; KCNA5; SLC30A3; RBPJ; CXCR4; CX3CL1; ARHGAP28; CLDN4; KDR; NPR2; PIM2; FGF13; IGFBP5; CXCL12; SQSTM1; PCSK1N; CCND1; VIM; ELOVL6; SPP1; CYP26A1; CMKLR1	4060	4060
1.87556604671e-05	0.000126646884869	GO:0006807	b	2	nitrogen compound metabolic process	595; 1240; 1364; 2043; 2249; 2258; 2766; 3488; 3516; 3791; 3958; 3992; 4882; 5577; 5787; 6376; 6595; 6660; 6696; 7078; 7431; 7481; 7852; 8091; 170690; 8878; 9252; 11040; 11221; 339896; 79071; 79923; 81848; 84803; 84891; 84969; 54796; 23608; 56999; 58524; 27344	DMRT3; TIMP3; EPHA4; GPAT3; MKRN1; LGALS3; PRKAR2B; ADAMTS16; GADL1; SMARCA2; SOX5; NANOG; RBPJ; ADAMTS9; CXCR4; CX3CL1; CLDN4; KDR; TOX2; NPR2; PIM2; HMGA2; SPRY4; FGF13; IGFBP5; FADS1; FGF4; DUSP10; GMPR; SQSTM1; PCSK1N; RPS6KA5; BNC2; CCND1; VIM; ELOVL6; SPP1; PTPRB; ZSCAN10; CMKLR1; WNT11	10378	10363

1.88623020017e-05	0.000126646884869	GO:0044238	b	2	primary metabolic process	595; 1240; 1364; 1592; 2043; 2249; 2258; 2766; 3488; 3516; 3791; 3958; 3992; 4882; 5577; 5787; 6376; 6595; 6660; 6696; 7078; 7431; 7481; 7852; 8091; 170690; 8878; 9252; 11040; 11221; 339896; 79071; 79923; 81848; 84803; 84891; 84969; 54796; 23608; 56999; 58524; 27344	DMRT3; TIMP3; EPHA4; GPAT3; MKRN1; LGALS3; PRKAR2B; ADAMTS16; GADL1; SMARCA2; SOX5; NANOG; RBPJ; ADAMTS9; TOX2; CXCR4; CX3CL1; CLDN4; KDR; PTPRB; NPR2; PIM2; HMGA2; SPRY4; FGF13; IGFBP5; FADS1; FGF4; DUSP10; GMPR; SQSTM1; PCSK1N; RPS6KA5; BNC2; CCND1; VIM; ELOVL6; SPP1; CYP26A1; ZSCAN10; CMKLR1; WNT11	10846	10831
3.31285014947e-05	0.00019063114593	GO:0042330	b	2	taxis	1240; 2043; 2249; 3791; 3958; 6376; 6387; 7852; 9252	CXCL12; RPS6KA5; EPHA4; CX3CL1; CXCR4; LGALS3; FGF4; CMKLR1; KDR	645	645
3.418562466e-05	0.00019063114593	GO:0019827	b	2	stem cell population maintenance	2249; 3516; 8091; 79923; 84891	NANOG; RBPJ; FGF4; HMGA2; ZSCAN10	145	145
3.50984723736e-05	0.00019063114593	GO:0051716	b	2	cellular response to stimulus	595; 1240; 1592; 2043; 2249; 2258; 3488; 3516; 3741; 3791; 3958; 3992; 4882; 5137; 5577;	TIMP3; EPHA4; GPAT3; LGALS3; PRKAR2B; SPRY4; KCNA5; SOX5; FGF4; NANOG; PDE1C;	7661	7653

						6376; 6387; 6660; 6696; 7078; 7431; 7481; 7852; 8091; 8878; 9252; 11040; 11221; 79923; 81848; 84803; 79822; 284339; 27344	RBPJ; CXCR4; CX3CL1; ARHGAP28; KDR; NPR2; PIM2; HMGA2; FGF13; TMEM145; IGFBP5; FADS1; CXCL12; DUSP10; SQSTM1; PCSK1N; RPS6KA5; CCND1; VIM; SPP1; CYP26A1; CMKLR1; WNT11		
3.65038364546e-05	0.00019063114593	GO:0098727	b	2	maintenance of cell number	2249; 3516; 8091; 79923; 84891	NANOG; RBPJ; FGF4; HMGA2; ZSCAN10	147	147
5.87198054992e-05	0.000282205356085	GO:0071704	b	2	organic substance metabolic process	595; 1240; 1364; 1592; 2043; 2249; 2258; 2766; 3488; 3516; 3791; 3958; 3992; 4882; 5577; 5787; 6376; 6595; 6660; 6696; 7078; 7431; 7481; 7852; 8091; 170690; 8878; 9252; 11040; 11221; 339896; 79071; 79923; 81848; 84803; 84891; 84969; 54796; 23608; 56999; 58524; 27344	DMRT3; TIMP3; EPHA4; GPAT3; MKRN1; LGALS3; PRKAR2B; ADAMTS16; GADL1; SMARCA2; SOX5; NANOG; RBPJ; ADAMTS9; TOX2; CXCR4; CX3CL1; CLDN4; KDR; PTPRB; NPR2; PIM2; HMGA2; SPRY4; FGF13; IGFBP5; FADS1; FGF4; DUSP10; GMPR; SQSTM1; PCSK1N; RPS6KA5; BNC2; CCND1; VIM; ELOVL6; SPP1;	11233	11218

							CYP26A1; ZSCAN10; CMKLR1; WNT11		
6.30458774 232e-05	0.00028220 5356085	GO:004887 0	b	2	cell motility	1240; 1364; 2043; 2249; 2258; 3488; 3791; 3958; 6376; 6387; 7481; 7852; 11221; 56999	CX3CL1; CXCL12; CXCR4; CMKLR1; EPHA4; ADAMTS9; LGALS3; WNT11; IGFBP5; FGF13; FGF4; CLDN4; DUSP10; KDR	1700	1698
6.30458774 232e-05	0.00028220 5356085	GO:005167 4	b	2	localization of cell	1240; 1364; 2043; 2249; 2258; 3488; 3791; 3958; 6376; 6387; 7481; 7852; 11221; 56999	CX3CL1; CXCL12; CXCR4; CMKLR1; EPHA4; ADAMTS9; LGALS3; WNT11; IGFBP5; FGF13; FGF4; CLDN4; DUSP10; KDR	1700	1698
7.75624677 401e-05	0.00033140 3271253	GO:004423 7	b	2	cellular metabolic process	595; 1240; 1364; 1592; 2043; 2249; 2258; 2766; 3488; 3516; 3791; 3958; 3992; 4882; 5577; 5787; 6376; 6595; 6660; 6696; 7078; 7431; 7481; 7852; 8091; 8878; 9252; 11040; 11221; 339896; 79071; 79923; 81848; 84803; 84891; 84969; 54796; 23608; 56999; 58524; 27344	DMRT3; TIMP3; EPHA4; GPAT3; MKRN1; LGALS3; PRKAR2B; SPRY4; GADL1; SMARCA2; SOX5; NANOG; RBPJ; ADAMTS9; TOX2; CXCR4; CX3CL1; CLDN4; KDR; PTPRB; NPR2; PIM2; HMGA2; FGF13; IGFBP5; FADS1; FGF4; DUSP10; GMPR; SQSTM1; PCSK1N; RPS6KA5; BNC2; CCND1; VIM;	10863	10848

							ELOVL6; SPP1; CYP26A1; ZSCAN10; CMKLR1; WNT11		
0.00011242 5569059	0.00045947 8412677	GO:004858 9	b	2	developmenta l growth	2258; 3516; 6387; 6696; 7481; 7852; 11221; 54796	BNC2; RBPJ; SPP1; CXCR4; FGF13; CXCL12; DUSP10; WNT11	587	587
0.00014058 3562628	0.00055061 8953626	GO:000971 9	b	2	response to endogenous stimulus	595; 1364; 2043; 2249; 3488; 3516; 5577; 6387; 6660; 6696; 7078; 7431; 81848	CXCL12; TIMP3; CCND1; RBPJ; EPHA4; VIM; SPRY4; PRKAR2B; SPP1; IGFBP5; FGF4; CLDN4; SOX5	1597	1597
0.00015116 0465094	0.00056836 3348753	GO:006500 9	b	2	regulation of molecular function	595; 1240; 1364; 2043; 2258; 3791; 3958; 5577; 5787; 6376; 7078; 7481; 7852; 8091; 9252; 11221; 81848; 79822; 27344	PCSK1N; ARHGAP28; TIMP3; WNT11; CCND1; CMKLR1; EPHA4; HMGA2; SPRY4; LGALS3; PRKAR2B; PTPRB; CXCR4; FGF13; CX3CL1; CLDN4; DUSP10; KDR; RPS6KA5	3142	3138
0.00017380 4112451	0.00062836 8714247	GO:001604 9	b	2	cell growth	2258; 3488; 6387; 6595; 6696; 7481; 7852	CXCR4; SPP1; IGFBP5; FGF13; SMARCA2; CXCL12; WNT11	466	466
0.00019026 8247228	0.00066241 5379239	GO:000726 7	b	2	cell-cell signaling	595; 2043; 2249; 2258; 3516; 3741; 3992; 5577; 6376; 6696; 7481; 8091; 8878	CX3CL1; KCNA5; SQSTM1; CCND1; RBPJ; EPHA4; HMGA2; FADS1; PRKAR2B; SPP1;	1646	1646

							FGF13; FGF4; WNT11		
0.00023925 7262694	0.00080322 0810472	GO:004887 1	b	2	multicellular organismal homeostasis	1240; 1364; 3516; 5577; 6696; 7852; 79071	CMKLR1; RBPJ; ELOVL6; PRKAR2B; SPP1; CXCR4; CLDN4	491	491
0.00046414 6070608	0.01067535 9624	GO:003054 5	m	2	receptor regulator activity	2249; 2258; 3958; 6376; 6387; 6696; 7481	CX3CL1; FGF4; LGALS3; SPP1; FGF13; CXCL12; WNT11	548	548
0.00063166 850837	0.00204747 723403	GO:199084 5	b	2	adaptive thermogenesis	1240; 3516; 7852; 79071	ELOVL6; RBPJ; CMKLR1; CXCR4	153	153
0.00084333 0396105	0.00264243 524113	GO:000695 0	b	2	response to stress	595; 1240; 1364; 2043; 2766; 3516; 3741; 3791; 3958; 3992; 5577; 6376; 6387; 6696; 7431; 7852; 8091; 8878; 9252; 11221; 27344	CX3CL1; PCSK1N; CXCL12; RPS6KA5; SQSTM1; CCND1; CMKLR1; RBPJ; EPHA4; HMGA2; VIM; FADS1; PRKAR2B; SPP1; KDR; CXCR4; LGALS3; KCNA5; CLDN4; DUSP10; GMPR	4192	4187
0.00095170 2546121	0.00288580 77205	GO:000715 5	b	2	cell adhesion	1364; 2043; 3791; 3958; 6376; 6387; 6696; 7852; 11221; 81848; 56999	EPHA4; ADAMTS9; SPRY4; CX3CL1; SPP1; CXCR4; LGALS3; CXCL12; CLDN4; DUSP10; KDR	1457	1456
0.00114304 13706	0.00335768 402614	GO:000905 8	b	2	biosynthetic process	595; 1240; 2249; 2766; 3488; 3516; 3791; 3992; 4882; 6376; 6595; 6660; 6696; 7431; 7481;	DMRT3; GPAT3; GADL1; SMARCA2; SOX5; NANOG; RBPJ; CX3CL1; KDR; NPR2; PIM2;	6288	6282

						8091; 8878; 9252; 11040; 339896; 79071; 79923; 84803; 84891; 84969; 54796; 58524	HMGA2; IGFBP5; FADS1; FGF4; GMPR; SQSTM1; RPS6KA5; BNC2; CCND1; VIM; ELOVL6; SPP1; TOX2; ZSCAN10; CMKLR1; WNT11		
0.00118648209477	0.00337967626994	GO:0001502	b	2	cartilage condensation	2249; 6660	FGF4; SOX5	20	20
0.00151276520932	0.0173967999071	GO:0030234	m	2	enzyme regulator activity	595; 2258; 3958; 5577; 7078; 7481; 81848; 79822; 27344	PCSK1N; TIMP3; CCND1; LGALS3; PRKAR2B; SPRY4; FGF13; ARHGAP28; WNT11	1084	1080
0.00304230534351	0.00841107947913	GO:0001503	b	2	ossification	3488; 3516; 4882; 6696; 7481	RBPJ; SPP1; NPR2; WNT11; IGFBP5	388	387
0.00308911650426	0.148277592204	GO:0031012	c	2	extracellular matrix	3958; 6387; 7078; 7481; 170690; 56999	TIMP3; ADAMTS9; ADAMTS16; LGALS3; CXCL12; WNT11	566	565
0.00314462099741	0.0084455535359	GO:0050673	b	2	epithelial cell proliferation	595; 3488; 3791; 6387; 11221	CCND1; CXCL12; DUSP10; KDR; IGFBP5	390	390
0.00331676856826	0.00851512669019	GO:0009628	b	2	response to abiotic stimulus	595; 2766; 3516; 3741; 6387; 7481; 7852; 8091; 27344	PCSK1N; CXCL12; CCND1; RBPJ; HMGA2; GMPR; CXCR4; KCNA5; WNT11	1213	1212
0.00335169880358	0.00851512669019	GO:0030534	b	2	adult behavior	2043; 6387; 58524	CXCL12; EPHA4; DMRT3	117	117
0.00352254428684	0.0270061728658	GO:0044877	m	2	protein-containing	595; 3791; 3958; 6376; 6387; 6696; 7431; 8091; 8878	SQSTM1; CCND1; HMGA2; VIM; CX3CL1; SPP1;	1223	1223

					complex binding		LGALS3; CXCL12; KDR		
0.00426276863518	0.010544743466	GO:0010463	b	2	mesenchymal cell proliferation	2249; 7481	FGF4; WNT11	38	38
0.00500294600707	0.0120583826837	GO:0061351	b	2	neural precursor cell proliferation	2258; 6376; 6660	FGF13; CX3CL1; SOX5	135	135
0.00521066429781	0.0122450610998	GO:0048646	b	2	anatomical structure formation involved in morphogenesis	3516; 3791; 5787; 7481; 7852; 8091; 79923; 56999	NANOG; ADAMTS9; RBPJ; HMGA2; KDR; PTPRB; CXCR4; WNT11	1063	1061
0.00569551304731	0.0130580055231	GO:0051707	b	2	response to other organism	3516; 6376; 6387; 7431; 7852; 8091; 11040; 11221	PIM2; RBPJ; HMGA2; VIM; CXCR4; CX3CL1; CXCL12; DUSP10	1078	1077
0.00646412940605	0.0144673372421	GO:0003006	b	2	developmental process involved in reproduction	595; 3516; 4882; 6595; 6696; 58524	NPR2; DMRT3; CCND1; RBPJ; SPP1; SMARCA2	658	658
0.00702298225319	0.0153525658558	GO:0009607	b	2	response to biotic stimulus	3516; 6376; 6387; 7431; 7852; 8091; 11040; 11221	PIM2; RBPJ; HMGA2; VIM; CXCR4; CX3CL1; CXCL12; DUSP10	1117	1116
0.00740007205627	0.0158092448475	GO:0090130	b	2	tissue migration	2249; 3791; 11221; 56999	FGF4; ADAMTS9; DUSP10; KDR	302	302
0.00758104282387	0.015835956121	GO:0003008	b	2	system process	1364; 2258; 3488; 3741; 4882; 5577; 6387; 7078; 7431; 7852; 170690; 58524	ADAMTS16; NPR2; CXCL12; TIMP3; CXCR4; VIM; PRKAR2B; DMRT3;	2176	2174

							IGFBP5; FGF13; KCNA5; CLDN4		
0.00840912169216	0.0171838573709	GO:0007626	b	2	locomotory behavior	2043; 6387; 58524	CXCL12; EPHA4; DMRT3	163	163
0.00869338088992	0.0173867617798	GO:0048771	b	2	tissue remodeling	3488; 3516; 6696	RBPJ; SPP1; IGFBP5	165	165
0.00875865480752	0.0409023045667	GO:0050840	m	2	extracellular matrix binding	3958; 6696	LGALS3; SPP1	55	55
0.00889180534058	0.0409023045667	GO:0140096	m	2	catalytic activity, acting on a protein	595; 2043; 3791; 4882; 5787; 170690; 8878; 9252; 11040; 11221; 23608; 56999	SQSTM1; NPR2; PIM2; RPS6KA5; CCND1; EPHA4; ADAMTS9; MKRN1; PTPRB; ADAMTS16; DUSP10; KDR	2220	2219

Supplementary Table 2. The primer sequences used for DNA sequencing and real-time PCR.

Gene symbol	Primer	Primer sequence (5'→3')	Product length
**Primers for DNA sequencing**			
ESX1	Forward	GGCCTACAAAAATAACAGGGCAT	830
	Reverse	TTCCCCTCTGAAGGTTTGGTT	
**Primers for Real-time PCR**			
ESX1	Forward	AACTTACCGTGACCTCGCTG	110
	Reverse	GGACCCTTCCGTGCCAAC	
CCND1	Forward	CCCTCGGTGTCCTACTTCAAAT	173
	Reverse	CTCTTTTTCACGGGCTCCAG	
HMGA2	Forward	AGCAGCAAGAACCAACCG	251
	Reverse	AGTGGCATTTTTAGAGAACAGTAGA	
L1TD1	Forward	CTTTGTTCGCTCCTCAGTCG	126
	Reverse	TTCATCCGCCTCTGTACCTTC	
LGALS3	Forward	CGGAGCCAGCCAACGAG	108
	Reverse	CCAGACCCAGATAACGCATCA	
MKRN1	Forward	CAGTGGGAGAGTGCCGATA	281
	Reverse	GAGACAGTAGGTGTGGTTGC	
NANOG	Forward	ACCAGTCCCAAAGGCAAACA	299

	Reverse	CATCCCTGCGTCACACCATT	
PDE1C	Forward	AGAACAGCAACAGAATGGTGAC	198
	Reverse	GTGGAAGAAGGAGAAGAAGGAGAA	
WNT11	Forward	GCATCAAGTGGCTGGCG	278
	Reverse	TGAGGGTGGGAGGGGAAG	
CXCR4	Forward	CAGCGTCTCAGTGCCCTTTT	111
	Reverse	AATCCTACAACTCTCCTCCCCA	
FGF4	Forward	GGGGCGTGGTGAGCATC	90
	Reverse	CGGTGAAGAAGGGCGAGC	
LINC00662	Forward	AAGGATGACTGGAGGGAGTTTC	135
	Reverse	GGCTTTTGGCTTTTCAATGGC	
RPS6KA5	Forward	TTGACTGGTGGAGTTTGGGTG	154
	Reverse	TTGGGGATATGGAGGCTCACT	
ACT	Forward	CTTCCTGGGCATGGAGTCC	192
	Reverse	ATCTTGATCTTCATTGTGCTG	
GAPDH	Forward	GCTCTCTGCTCCTCCTGTTC	112
	Reverse	ACCAAATCCGTTGACTCCGA	
